# A new method for ecologists to estimate heterozygote excess and deficit for multi‐locus gene families

**DOI:** 10.1002/ece3.11561

**Published:** 2024-07-23

**Authors:** Gabe D. O'Reilly, Oliver Manlik, Sandra Vardeh, Jennifer Sinclair, Belinda Cannell, Zachary P. Lawler, William B. Sherwin

**Affiliations:** ^1^ Evolution and Ecology Research Centre, School of Biological Earth and Environmental Science University of New South Wales Sydney New South Wales Australia; ^2^ Department of Bioinformatics University of North Carolina at Charlotte Charlotte North Carolina USA; ^3^ Biology Department United Arab Emirates University Al Ain, Abu Dhabi UAE; ^4^ Bundesamt für Naturschutz Bonn Nordrhein‐Westfalen Germany; ^5^ Cape Bernier Vineyard Bream Creek Tasmania Australia; ^6^ Oceans Institute/School of Biological Sciences University of Western Australia Crawley Western Australia Australia; ^7^ School of Environmental and Conservation Sciences Murdoch University Murdoch Western Australia Australia; ^8^ The University of Newcastle Newcastle New South Wales Australia

**Keywords:** *F*_IS_, fixation index, inbreeding, population genetics, selection, simulation

## Abstract

The fixation index, *F*_IS_, has been a staple measure to detect selection, or departures from random mating in populations. However, current Next Generation Sequencing (NGS) cannot easily estimate *F*_IS_, in multi‐locus gene families that contain multiple loci having similar or identical arrays of variant sequences of ≥1 kilobase (kb), which differ at multiple positions. In these families, high‐quality short‐read NGS data typically identify variants, but not the genomic location, which is required to calculate *F*_IS_ (based on locus‐specific observed and expected heterozygosity). Thus, to assess assortative mating, or selection on heterozygotes, from NGS of multi‐locus gene families, we need a method that does not require knowledge of which variants are alleles at which locus in the genome. We developed such a method. Like *F*_IS_, our novel measure, ^1^ *H*_IS_, is based on the principle that positive assortative mating, or selection against heterozygotes, and some other processes reduce within‐individual variability relative to the population. We demonstrate high accuracy of ^1^ *H*_IS_ on a wide range of simulated scenarios and two datasets from natural populations of penguins and dolphins. ^1^ *H*_IS_ is important because multi‐locus gene families are often involved in assortative mating or selection on heterozygotes. ^1^ *H*_IS_ is particularly useful for multi‐locus gene families, such as toll‐like receptors, the major histocompatibility complex in animals, homeobox genes in fungi and self‐incompatibility genes in plants.

## Graphical abstract

The fixation index, *F*_IS_, has been a staple measure to assess assortative mating or selection on heterozygotes. However, current Next Generation Sequencing (NGS) cannot easily determine which variants are allelic at which locus—a requirement to calculate *F*_IS_. We have developed a method called H1IS to assess assortative mating, or selection on heterozygotes, from NGS of multi‐locus gene families, that does not require knowledge of which variants are allelic at which locus in the genome.

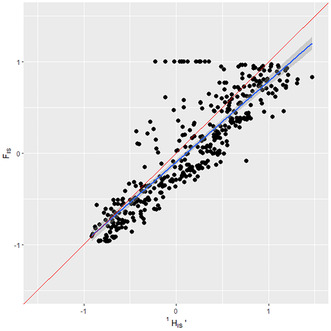

## 1 INTRODUCTION

The inference of assortative mating, selection and demographic processes in populations is the primary aim of much population genetics research because such knowledge can guide us to effectively manage populations. There are numerous methods to quantify indicators for positive and negative assortative mating (the latter including inbreeding), selection and demographic processes, but *F*_IS_ is the method that has seen the most use and is generally the standard (Crow & Kimura, 1970; Halliburton, 2004; Hedrick, 2005; Wright, 1950). *F*_IS_ is often called the inbreeding coefficient, but *F*_IS_ also has other applications that are not related to inbreeding or assortative mating (described below). *F*_IS_ is based on the analysis of variants at a particular locus or location in the genome; these variants at one location are called alleles. Alleles can be defined by a single nucleotide difference from other alleles at that location (single nucleotide polymorphism (SNP)) or can be haplotypes with multiple nucleotides differing between alternative alleles at the same location, the latter being the types of alleles considered in this article. Note that throughout this article, we use ‘variants’ to refer to any DNA sequences that differ from one another, irrespective of whether they are at the same locus in the genome (and thus are ‘alleles’) or are at different loci in a multi‐locus gene family.

*F*_IS_ compares the expected proportion of heterozygotes, based on Hardy–Weinberg equilibrium in a randomly mating population ($H_{e}$, Equation 1), to the actual number of heterozygotes observed in a study population ($H_{o}$, Equation 2). $H_{e}$ is calculated from the proportions of alleles in the population and is commonly used as a measure of genetic diversity (Halliburton, 2004):

(1) $$H_{e}=1-\sum_{i=1}^{\tilde{V}}{\tilde{P_{i}}}^{2}$$

where capital ‘$\tilde{V}$’ is the number of variants in the population (in this case, allelic types) for a particular locus and $\tilde{P_{i}}$ is the proportion of the *i*th allele in the population ($\sum_{i=1}^{\tilde{V}}\tilde{P_{i}}=1$) (Halliburton, 2004). Note that the tilde (~) indicates values for a single locus, to distinguish these from the multi‐locus values used in most of this article.

(2) $$H_{o}=\text{Proportion of population that is heterozygous}\;\mathrm{at}\;\text{that locus}$$

Again, using locus‐specific values, the equation for *F*_IS_ is (Halliburton, 2004)

(3) $$F_{\text{IS}}=\frac{H_{e}-H_{o}}{H_{e}}$$

Note that we do not place a tilde over $H_{e}$ or $H_{o}$, because these values must always be locus‐specific, so there is no need to distinguish them from non‐locus‐specific values. This comparison gives an *F*_IS_ value between −1 and +1 that indicates how the number of heterozygotes in the population deviates from what is expected under random mating conditions. A positive *F*_IS_ value indicates that there are fewer heterozygotes than expected under Hardy–Weinberg equilibrium expectations, including random mating. For instance, a population with positive assortative mating (i.e., mating with genetically similar individuals, including inbreeding) will often have a much lower proportion of heterozygotes than expected, and this deficit leads to a positive *F*_IS_ value; in the extreme case when no heterozygotes are observed at all, despite available allelic variation, then *F*_IS_ = +1 (Table 1). In contrast, a negative *F*_IS_ value will result from higher proportion of heterozygotes than expected under random mating, also called heterozygote excess (Table 1). This excess of heterozygotes might be caused by negative assortative mating—choice of genetically dissimilar mates. Thus, *F*_IS_ for selectively neutral genes can be used to infer negative and positive assortative mating. However, as well as the effects of assortative mating, the heterozygote excess or deficit that *F*_IS_ measures can also be due to selection for or selection against heterozygous individuals. Excess or deficit of heterozygotes can also be caused by other factors such as unusual chromosomal arrangements (which we do not discuss here, except for some mention of autopolyploidy) or the Wahlund effect (Halliburton, 2004). Thus, if independent data show that the population is isolated and randomly mating, *F*_IS_ might be useful for detecting signatures of selective pressures for or against heterozygous individuals (Crow & Kimura, 1970; Halliburton, 2004; Hedrick, 2005).

**TABLE 1:** Comparison of *F*_IS_ and our new method (^1^ *H*_IS_).

	*F*_IS_	^1^ *H*_IS_
How many loci can it be used on?	One at a time	More than one simultaneously
Do you need to know which variants are at which locus in the genome?	Yes	No
Can it be used on multi‐locus gene families with NGS data?	No	Yes
Value resulting from homozygote excess (e.g., from positive assortative mating such as inbreeding or from selection against heterozygotes)	Positive value (+)	Positive value (+)
Value reflecting homozygote deficit (e.g., from negative assortative mating or from selection for heterozygotes)	Negative value (−)	Negative value (−)
Range of values	−1 to +1	−1 to ~+1

*F*_IS_ can be calculated by two methods: either for a single locus or averaged over multiple un‐linked/independent loci that do not share common alleles (Halliburton, 2004; Hedrick, 2005). To calculate *F*_IS_ on multiple loci, first *F*_IS_ is calculated independently at each locus, then the arithmetic mean is taken across those loci to get a single *F*_IS_ value. This method is often called ‘Multiple *F*_IS_’. It is possible to do such calculations with very poor data (Vieira et al., 2016), but this method does not address the problem of multi‐locus gene families.

Specifically, the problem we address in this article is that *F*_IS_ and Multiple *F*_IS_ are difficult to derive from multi‐locus gene families, because these families can share variants across several loci, either adjacent or scattered through the genome (Ellis et al., 2005; Zagalska‐Neubauer et al., 2010). For instance, genes of the major histocompatibility complex (MHC) and toll‐like receptors (TLRs) are often located in close proximity and have recently undergone duplication events, resulting in allelic polymorphism shared between loci (Goebel et al., 2017; Kulski et al., 2002; Liu et al., 2019; Velová et al., 2018). Note that we cited studies on both MHC and TLR multi‐locus gene families here. Unfortunately, for such multi‐gene families, it is often problematic to discern which variants are ‘allelic’, i.e., whether they are present at the same locus in the genome (Vekemans et al., 2021). These variants, which are a kilobase or longer, are typically not biallelic, but contain multiple SNP differences between pairs of variants. Multi‐locus gene families are of particular interest when investigating possible assortative mating patterns and selection on heterozygotes (Sommer, 2005). For example, multi‐locus gene families such as the MHC have been associated with fitness and various fitness components; MHC can mediate immune defence (Altizer et al., 2003; Klein, 1986) and reproductive success (Kalbe et al., 2009; Sepil et al., 2013; Thoss et al., 2011). MHC genes may also be associated with assortative mating, though this is still debated (Radwan et al., 2020). These potential associations make MHC an important multi‐locus gene family to study for examining assortative mating and selection on heterozygotes.

**FIGURE 1:**
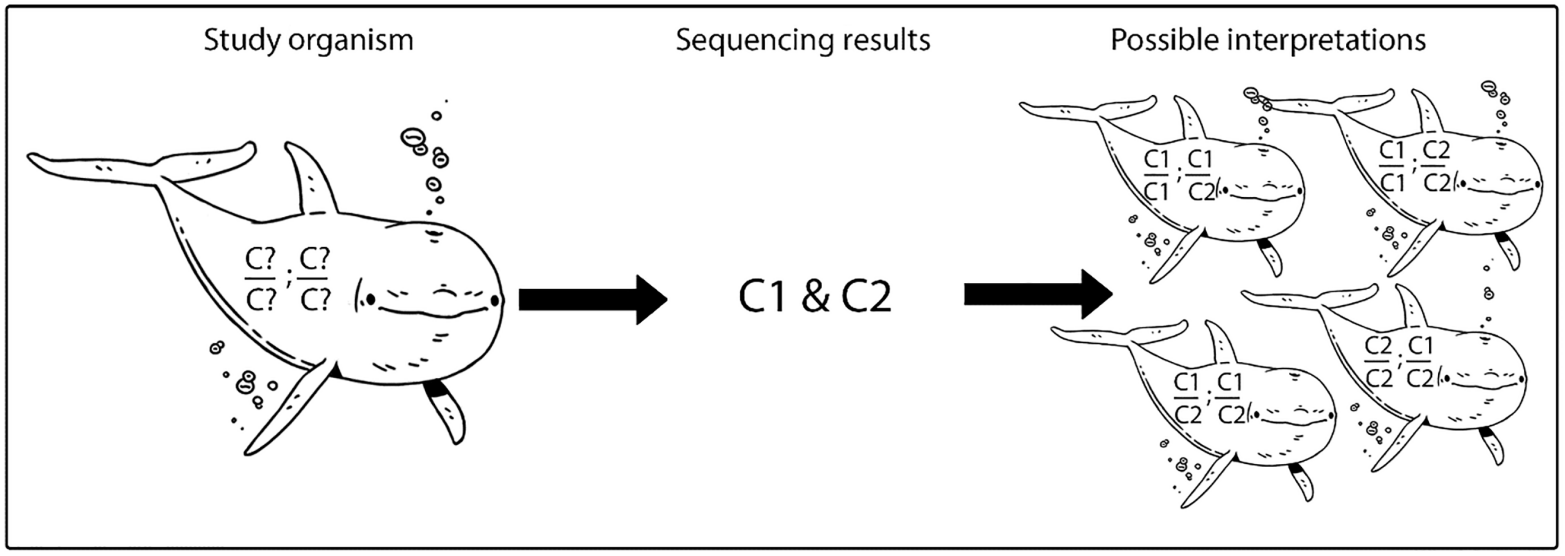
A schematic drawing of an oversimplified, hypothetical case showing the results from sequencing a multi‐locus gene family, which unusually, only has two unlinked loci each with only two alleles. Results are shown for an individual (variants C1 and C2), demonstrating the ambiguity those results can give even in such an oversimplified case. Each locus is shown as the two alleles at that genomic position in that individual, one allele above the other; the semicolon ‘;’ shows that it is unknown whether the loci are adjacent in the genome. *Note*: While this figure shows a situation with two loci and two variants, this paper investigates situations with many more loci and variants.

Even with model species, it can be difficult to determine how many loci are there, and which variants are alleles at the same location (locus) in the genome (Babik, 2010). Generally, the sequencing output will just give relative abundance of each variant per individual summed over all loci at which the variants appear, as is shown in an oversimplified case in Figure 1 (Manlik, 2016; Vardeh, 2015). This problem continues: for multi‐locus gene families, Next Generation Sequencing is still very poor at providing information on allelism (i.e., which variant sequences are segregating at which loci) (Roved et al., 2022), despite the fact that allelism is essential information for deriving *F*_IS_. Even with new specific software, allelism cannot be reliably determined in the most commonly analysed multi‐locus gene family (MHC), without addition of extensive multi‐generation pedigrees (Roved et al., 2022), which of course are unattainable in most species. Even with such pedigrees, the information only ‘increases the likelihood that segregation patterns of common alleles can be resolved’; in other words, the allelism of many variants would not be resolved (Roved et al., 2022). As shown in Figure 1, there is considerable ambiguity, even with the simplest possible multi‐locus gene family containing only two loci and two variant sequences. With such ambiguity, it becomes challenging to accurately calculate $H_{e}$ or $H_{o}$ per locus, and therefore it is not reasonable to calculate *F*_IS_ without making numerous assumptions such as: which variants are alleles at which locus, or that all SNPs are in Hardy–Weinberg equilibrium. Figure 1 also depicts another assumption that we would have to make to calculate *F*_IS_ with the conventional method—we need to know exactly how many loci make up the multi‐locus gene family—however, outside of model organisms or heavily studied multi‐locus gene families, this is often not the case. Not knowing the exact number of loci adds even more ambiguity to the calculation of *F*_IS_. These obstacles are compounded in the common case that the gene family contains more than two loci and more than two variant sequences (Ellis et al., 2005; Sommer, 2005; Zagalska‐Neubauer et al., 2010). Those authors point to difficulties due to either sequencing errors causing incorrect splitting or lumping of variant classes and/or mapping errors causing uncertain location and co‐location of variants; the latter being more important for short‐read NGS data and the former being more important for long‐read sequencing. Note that in Figure 1 (and in many cases of real data), we do not know whether the multiple loci are adjacent or scattered through the genome.

*F*_IS_ is also difficult to calculate when analysing autopolyploid species that contain more than two homologues of each chromosome. Of course, autopolyploidy leads to multi‐locus gene families, and again, sometimes researchers do not know how many homologue chromosomes are there. We will refrain from referring to this autopolyploid issue directly for the rest of the paper, but all the solutions we apply to multi‐locus gene families can be applied in the same manner to autopolyploid data.

This paper aims to devise an adequate solution to the problem of calculating an *F*_IS_ equivalent in multi‐locus gene families from NGS data, which we denote ^1^ *H*_IS_ (see Table 1). We develop an equation to estimate departure from Hardy–Weinberg equilibrium, which, like *F*_IS_, can be used in applications such as assessing a population's positive or negative assortative mating, or selection on heterozygotes, within multi‐locus gene families. We focus on the typical case of more than two loci in the gene family, and many more than two variants shared between these loci.

## 2 MATERIALS AND METHODS

### 2.1 Equation

Our method is based on the rationale that when there is either positive assortative mating (including inbreeding) or selection against heterozygotes, there is expected to be less diversity of variants within each individual relative to the total diversity of variants across the population. The opposite is true of populations that experience negative assortative mating (including outbreeding) or selection for heterozygotes. The total amount of diversity an individual can hold is also linked to the number of loci present. The method described below is based on these understandings, and with them we can construct an equation for assessing heterozygote deficits or excesses for multi‐locus gene families, by applying Shannon's information theory to the problem. Other approaches were attempted; however, they did not give suitable results (Supplement S1, Figures S1–S3). Shannon's information (H1) is a general measure of diversity, originally developed for telecommunications (Shannon, 1949), and since applied to population genetics (Manlik, Chabanne, et al., 2019; O'Reilly et al., 2020; Sherwin et al., 2017, 2021). A potential *F*_IS_ analogue based on Shannon's information compares the diversity of variants within each individual to the total diversity of variants across the population:

(4) H′IS1=−HI1¯L+1HS1−L

where the number of loci is *L* (or one of the three possible estimates of *L* is used, see Supplement S4), and HI1 is the Shannon's information per individual based on the proportions seen within each individual's NGS data for each variant $p_{i}$, using the equation HI1=−∑i=1vpilnpi. Lower case ‘*v*' is the total number of variants in the individual (that may or may not be alleles at the same locus $\sum_{i=1}^{v}p_{i}=1$). Then to produce HI1¯ one averages those Shannon's information values across all individuals to get HI1¯. HS1 is based on using the total proportions of variants in the whole population $P_{i}$ in the NGS dataset, to calculate Shannon's information as HS1=−∑i=1VPilnPi, where capital ‘*V*' is the total number of variants in the NGS dataset (that may or may not be alleles at the same locus $\sum_{i=1}^{V}P_{i}=1$).

In Equation 4, the foundation of HIS1 is the comparison between the diversity held within individuals (HI1¯) and the diversity held within the total population (HIS1), which is why Equation 4 takes the form of HI1¯HS1. When sampled individuals contain all the diversity found in the total population HI1¯HS1=1, indicating a high likelihood of heterozygotes, so we would expect a negative value of *F*_IS_, and thus we aim to derive an equation that gives a negative value associated with heterozygote excess. Additionally, unlike HS1, the maximum value of HI1 is dependent on L, whereas the maximum number of variants per individual is limited to 2L. Therefore, we use the number of loci to weight HI1 to L, making the calculation more sensitive to differences between HI1¯ and HS1, with more loci helping to differentiate between cases where low HI1¯ is due to a limit of maximum entropy ($\ln\left(2L\right)$) or just multiple copies of the same allele within an individual. For example: in a scenario where HI1¯=0.69 and HS1=1, with only two loci H′IS1=−0.07, close to 0 excess of deficit of homozygotes. With five loci, however, H′IS1=0.86, indicating an excess of homozygotes; with more loci, HI1 is more likely to be closer to HS1 if a population is in panmixia, because low HI1¯ will not be due to a limit of maximum entropy ($\ln\left(2L\right)$). With more loci, the numerator in the equation is inflated, possibly giving HI1¯L+1HS1 values greater than 1, which is then brought back to the −1 to +1 scale by −L. This puts H′IS1 on the same scale as FIS, with +1 indicating extreme deficit of heterozygotes, −1 indicating extreme excess of heterozygotes and zero indicating conformity to Hardy–Weinberg expectations (Table 1). Equation 4 is transformed into Equation 5, by adding a correction using the genetic evenness of the population.

(5) HIS1=−HI1¯L+1HS1−LEV

Genetic evenness ($E_{V}$) is a measure of how evenly distributed are alleles, with $E_{V}$ reaching its maximum value when all alleles are equally frequent, where Max HS1 = $\ln\left(V\right)$. So EV=HS1/lnV, where *V* is the number of variants in the population. With the same positive or negative assortative mating or selection, a more even distribution of variants in the population (greater evenness) would bring HI1¯ closer to HS1, depressing HIS1, so the multiplication by evenness corrects for this effect. For example: if HI1¯=0.69 and HS1=1, and L=5, then with max evenness ($E_{V}=1$), HIS1=0.86. However, if $E_{V}=0.1$ (the population is dominated by a single allele), HIS1=0.09, because a lower value for individuals (HI1¯) would be expected, as one allele would be much more common than any other. Equation 5 is used for HIS1 for the rest of this paper unless stated otherwise, because Equation 5 gave more accurate results when compared to *F*_IS_.

We demonstrate the general relationship between Equation 5 and *F*_IS_ for multiple loci and variants by simulations. Note that individual loci in multi‐locus families often have more than two alleles (haplotypes) per locus, so the remainder of the article deals with this multiallelic (multi‐haplotypic) case; extension to the rare case of multi‐locus families with biallelic loci would require additional work. Although HIS1=+1 is considered to indicate the maximum amount of positive assortative mating, it should be noted that, due to stochasticity, some simulation results have given values that go slightly above +1. We propose that in these cases, the result should be considered the same as if the value was just +1.

### 2.2 Creating a simulated dataset

In simulations, our goal was an even spread of *F*_IS_ values from −1 to 1, allowing us to investigate the relationship between HIS1 and *F*_IS_, over the entire range of possible values. Note that because our HIS1 method does not rely in any way upon information about the origin of the HIS1 and *F*_IS_ values, it was not important to model every possible underlying mechanism, including every type or strength of selection, or every possible mating pattern. Stochastic forward time simulations were performed on a wide range of scenarios chosen to provide an even spread of *F*_IS_ values from −1 to 1. We used the PYTHON package simuPOP v1.1.8 (Peng & Kimmel, 2005). Our simulations allowed 10 different variants per locus. Mutation did not occur and the loci were unlinked. The following parameters were varied to make up a wide range of scenarios:

1. Assortative mating: Positive (Inbred Small Family) and zero (Random Mating), with expectations of positive and zero $F_{\text{IS}}$, respectively
2. Selection: Mild Selection for Homozygotes (60% chance for homozygotes to reproduce, 50% chance for heterozygotes to reproduce); Mild Selection for Heterozygotes (20% chance for homozygotes to reproduce, 60% chance for heterozygotes to reproduce); and Strong Selection for Heterozygotes (0% chance for homozygotes to reproduce, 100% chance for heterozygotes to reproduce); with expectations of positive, mildly negative and strongly negative $F_{\text{IS}}$, respectively. Note that all selection scenarios were applied only to random mating populations, and also note that we did not include Strong Selection (100%) for homozygotes because such selection results in an unstable equilibrium, which quickly deteriorates into fixation (loss of all but one allele at each locus), in which case an analysis of HIS1 or $F_{\text{IS}}$ would be irrelevant.
3. Number of loci: 3 loci, 5 loci and 10 loci
4. Variant distribution: two extremely contrasting scenarios of variant proportions being Even (variants have equal proportions in the population) and Uneven (variants have a Poisson distribution, mean = 0.1);with 10 variants per locus in each case. Each variant can be present at any locus
5. Generations: 10 generations, 30 generations and 50 generations
6. Population size: 40 individuals, 400 individuals

All combinations of values of the parameters were tested (except without combining Assortative mating and Selection scenarios), giving a total of 180 scenarios, allowing us to investigate the full range of *F*_IS_ (−1 to +1). There were 100 replicates of each scenario. The data from these simulations were first used to calculate multiple *F*_IS_ for these simulated populations, because we knew the exact number of loci and which variants were allelic to them. Next, the data were converted to a format that resembled data with all the limitations of a real study (i.e., no information on which variants were at which loci, unknown number of loci), and Equation 5 was applied. Because the number of loci (*L*) was unknown in this modified dataset, we estimated the number of loci using the method one (‘One individual’ method) in Supplement S4. For our simulated dataset, we were able to assume our proportion of reads was an accurate representation of the variant distribution in the population—an assumption that may not be true for the real‐life datasets outlined in later sections. This is not an unusual assumption, for example, note that *F*_IS_ calculation from single loci also routinely assumes that the proportion of alleles in the sample is representative of the proportions in the sampled population. For the simulated dataset, there were no missing variants in the data (i.e., if an individual actually had a particular variant, that variant always appeared in the data). Additional simulations were also run to investigate the impact of read depth on HIS1 (Supplement S2).

### 2.3 Assessment of simulation results

Simulated data results were assessed by comparison of the HIS1 measurement to the $F_{\text{IS}}$ measurement of the same population. These comparisons were done with linear regression of $F_{\text{IS}}$ on HIS1, as well as by calculating Root Mean Squared Error (RMSE, Equation 6).

(6) RMSE=∑i=1nHIS1−FIS2n

where *n* is the number of values.

The data were filtered to remove any datum with no variation in the population (for example, where there was total fixation to a single variant at all loci). Values of *F*_IS_ were then binned at intervals of 0.1 from an *F*_IS_ range of −1.05 to +1.05. This binning, plus random selection of the same number of datapoints from each bin, ensures that the regression results would not be impacted by the large number of results close to *F*_IS_ = 0 in our dataset. Regression results without this binning can be found in Supplement S3.

### 2.4 Dolphin and penguin data

In addition to simulations, we also applied our method to real populations. We have applied these methods to MHC class I data from two dolphin populations (*Tursiops* sp.), Shark Bay (SB) and Bunbury (BB) (Manlik, 2016; Manlik et al., 2016); as well as to MHC class II data from three penguin populations (*Eudyptula minor*), Perth (PER), Albany (ALB) and Esperance (ESP) (Vardeh, 2015). We have also compared these results of the dolphin and penguin MHC data to *F*_IS_ results of microsatellite data (Manlik, Chabanne, et al., 2019; Vardeh, 2015) from those same populations, as a partial verification of the results of Equation 5. The proportion of microsatellite genotypes is affected by inbreeding, but it is unlikely to be affected by selection, therefore making microsatellite results useful to identify whether any results for MHC might be due to inbreeding. Additionally, *F*_IS_ was also derived from what appeared to be a single‐locus MHC dataset of 75 female dolphins from SB, using *MHC II* DQB (Manlik, Chabanne, et al., 2019). The full data processing, filtering and demographic information of the populations can be found in Manlik et al. (2016) for the dolphins and Vardeh (2015) for the penguins.

We assessed whether there is a heterozygote excess or deficit relative to Hardy–Weinberg equilibrium, which could be an indicator for non‐random mating, positive or negative selection, but also other factors such as a Wahlund effect. Manlik et al. (2016) suggested the MHC genes were under selection, and the microsatellite loci were thought to be neutral because they showed no significant departures from Hardy–Weinberg equilibrium. This should be kept in mind, because if these assumptions are true, they would be expected to cause discrepancy between HIS1 values of MHC and *F*_IS_ values of microsatellites—however, assortative mating and demographic (non‐selection) processes should affect both ^1^ *H*_IS_ values of MHC and *F*_IS_ values of microsatellites similarly. Unlike the simulated data, we did not know the exact number of loci for each multi‐locus gene family studied. Therefore, we estimated the number of loci for the penguins and dolphins using methods 1–3 in Supplement S4.

## 3 RESULTS

### 3.1 Simulated dataset results

**FIGURE 2:**
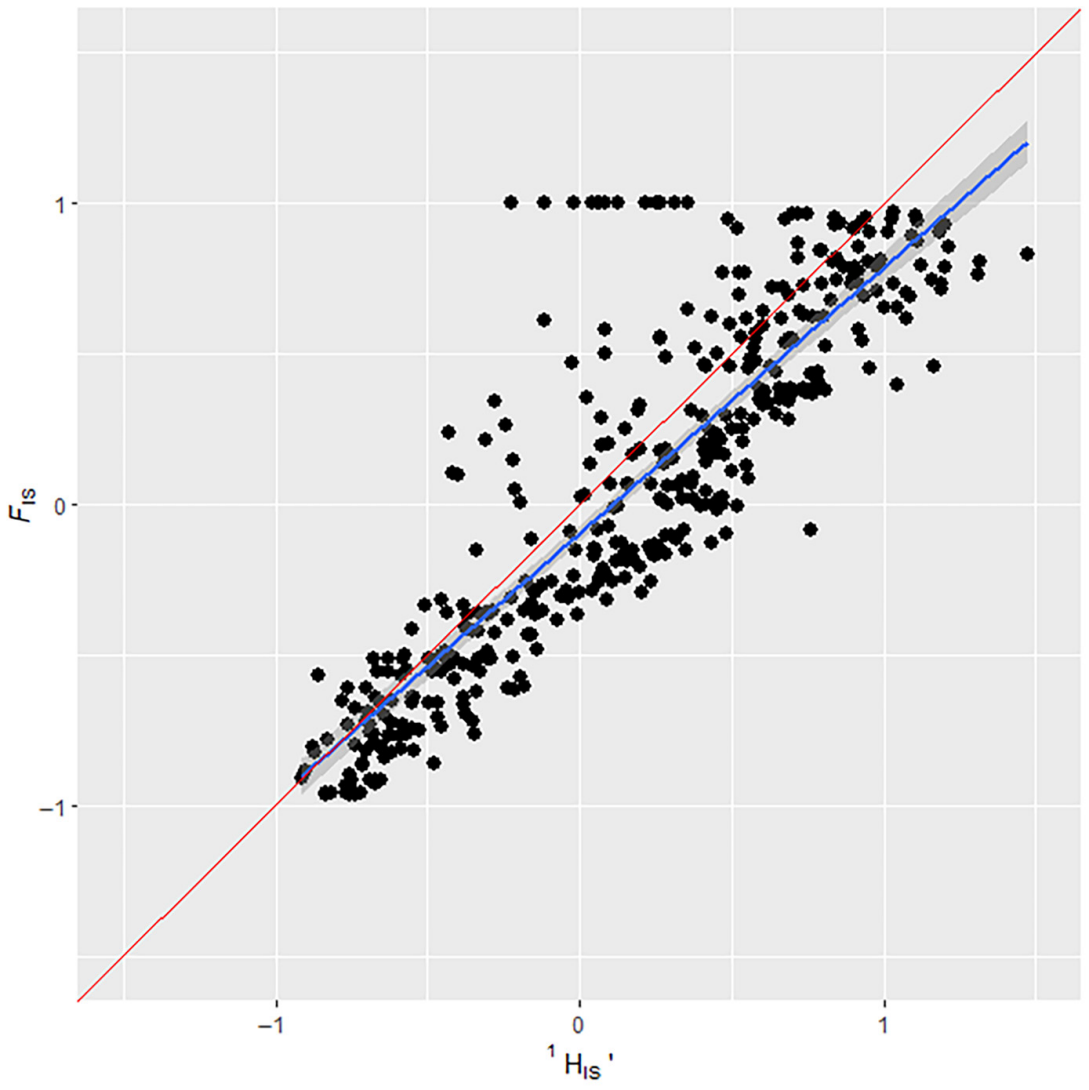
Regression of $F_{\text{IS}}$ on HIS1 in simulated data where replicates with no HI1 variation have been removed. $F_{\text{IS}}$ ranges were manipulated via ‘assortative mating’ and ‘selection’ scenario parameters shown in the methods section. The total binned data, with all scenarios together, are shown. Blue line indicates a regression slope with shading showing 95% confidence limits, the Red line indicates the expected 1:1 slope for perfect agreement between the methods. Regression analysis showed an *R*‐squared of .756, *p* = <.001 and RMSE = 0.398. Non‐binned data can be found in the supplement (Figure S6).

**FIGURE 3:**
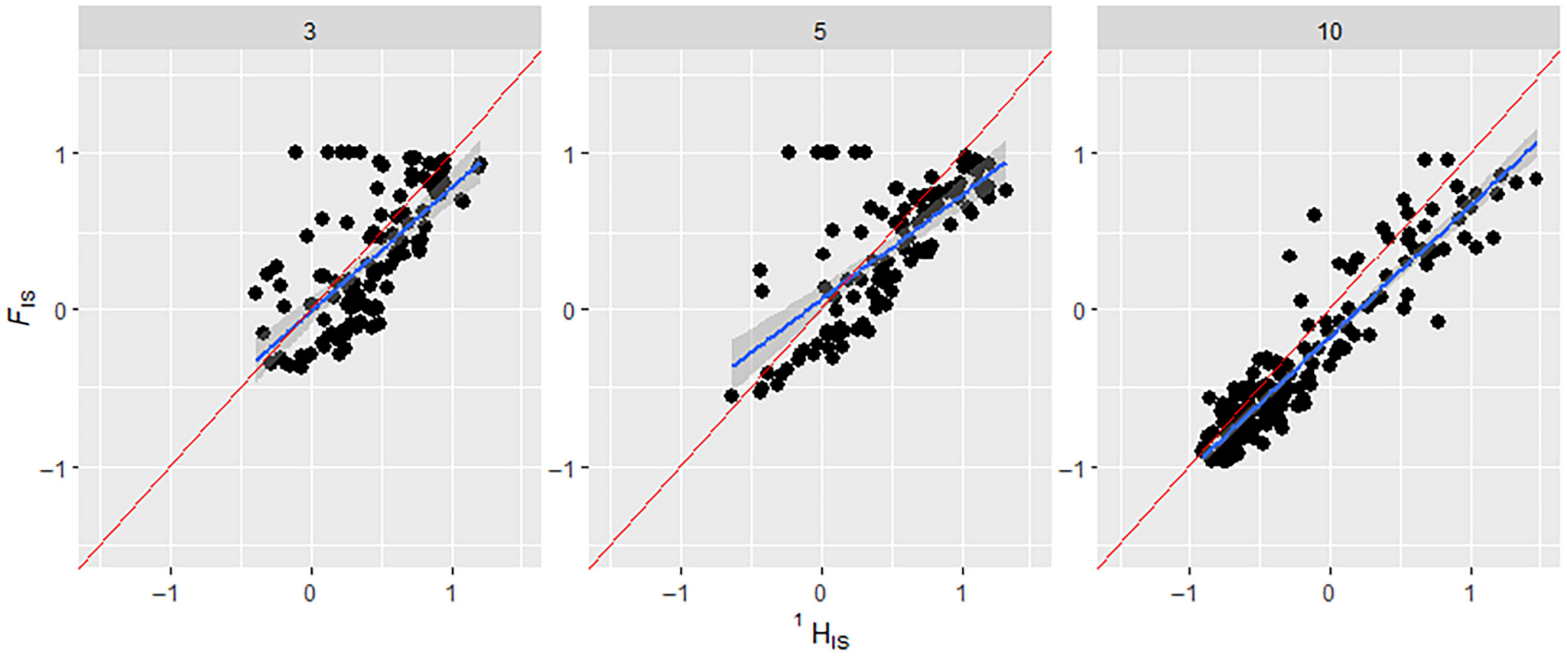
How the number of loci affects the regression of $F_{\text{IS}}$ on HIS1. Comparison of HIS1 results to their corresponding $F_{\text{IS}}$ results from simulated binned data that have had replicates with low HI1 variance removed. The $F_{\text{IS}}$ ranges were manipulated via ‘assortative mating’ and ‘selection’ scenario parameters shown in the methods section. The three panels show scenarios with differing numbers of loci set up in the simulation, indicated above each panel. Blue line indicates a regression slope, the Red line indicates the expected 1:1 slope. In scenarios with three loci, HIS1 showed an *R*‐squared of .445, *p*‐value = <.05 and RMSE = 0.334. In scenarios with five loci, HIS1 showed an *R*‐squared of .452, *p*‐value = <.05 and RMSE = 0.368. In scenarios with 10 loci, HIS1 showed an *R*‐squared of .861, *p*‐value = <.05 and RMSE = 0.255. Non‐binned data can be found in the supplement (Figure S7).

**FIGURE 4:**
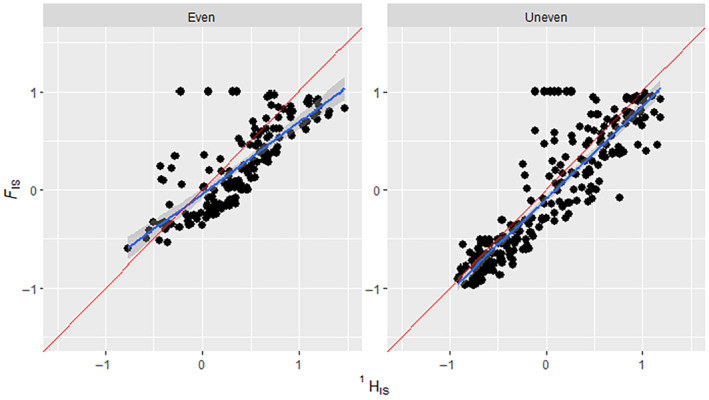
How the allele variant distribution affects the regression of $F_{\text{IS}}$ on HIS1. Comparison of HIS1 results to their corresponding $F_{\text{IS}}$ results from simulated binned data that have had replicates with low HI1 variance removed. The $F_{\text{IS}}$ ranges were manipulated via ‘assortative mating’ and ‘selection’ scenario parameters shown in the methods section. The two panels show scenarios with differing distribution of variants in the simulation, indicated above in each panel. Blue line indicates a regression slope, the Red line indicates the expected 1:1 slope. In scenarios with an Even variant distribution, HIS1 showed an *R*‐squared of .593, *p*‐value = <.05 and RMSE = 0.333. In scenarios with an Uneven variant distribution, HIS1 showed *R*‐squared of .795, *p*‐value = <.05 and RMSE = 0.300. Non‐binned data can be found in the supplement (Figure S8).

**FIGURE 5:**
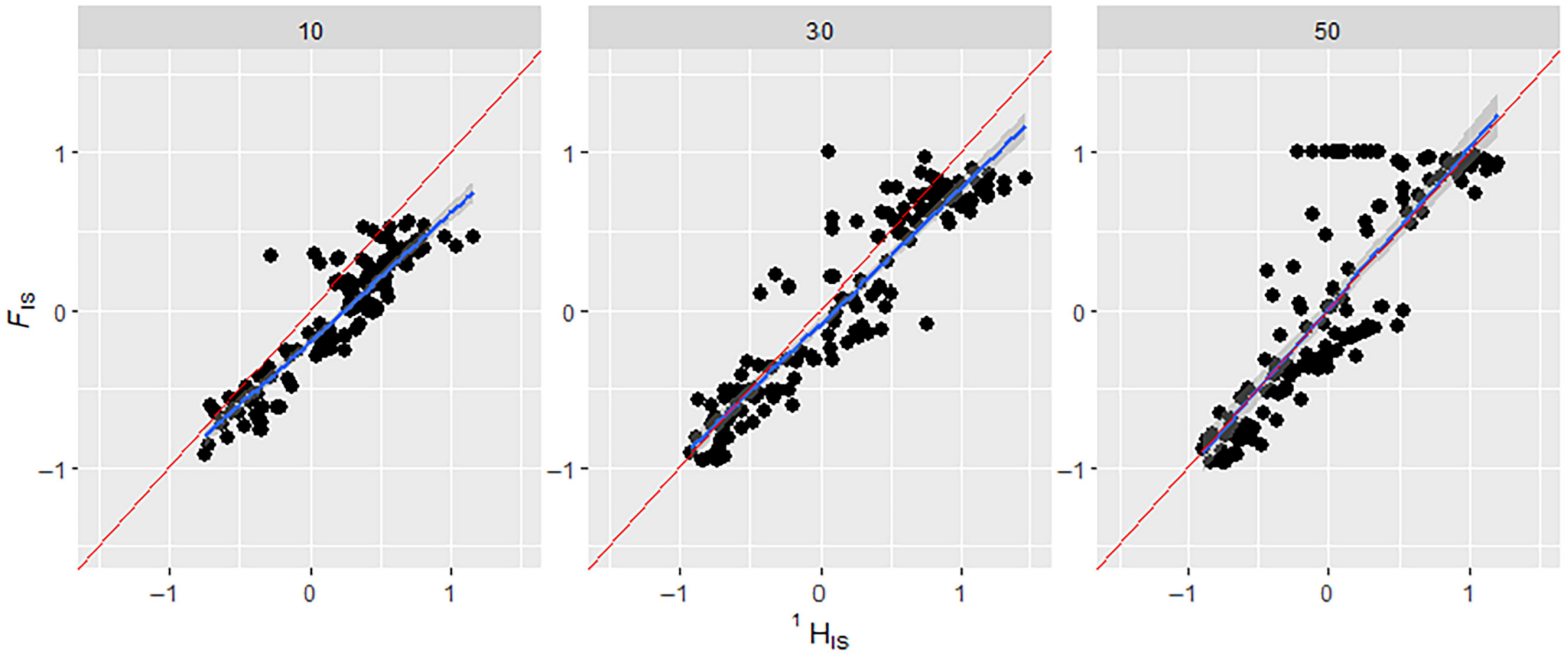
How the number of generations simulated affects the regression of $F_{\text{IS}}$ on HIS1 comparison. Comparison of HIS1 results to their corresponding $F_{\text{IS}}$ results from simulated binned data that have had replicates with low HI1 variance removed. The $F_{\text{IS}}$ ranges were manipulated via ‘assortative mating’ and ‘selection’ scenario parameters shown in the methods section. The three panels show scenarios with differing numbers of generations simulated, indicated above in each panel. Blue line indicates a regression slope, the Red line indicates the expected 1:1 slope. Ten‐generation data had an *R*‐squared of .827, *p*‐value = <.05 and RMSE = 0.299. Thirty‐generation data had an *R*‐squared of .855, *p*‐value = <.05 and RMSE = 0.273. Fifty‐generation data had an *R*‐squared of .723, *p*‐value = <.05 and RMSE = 0.362. Non‐binned data can be found in the supplement (Figure S9).

**FIGURE 6:**
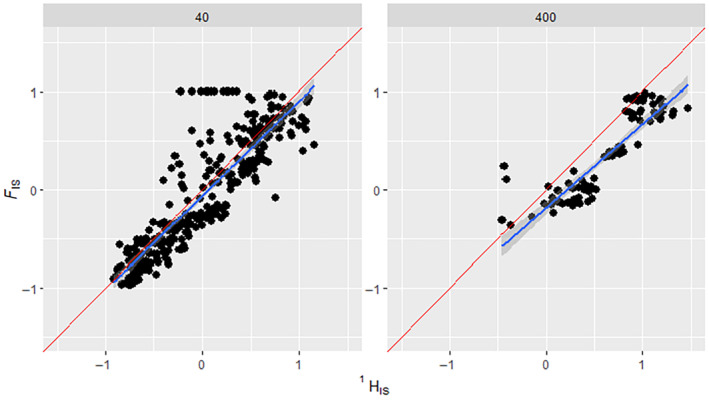
How population size affects the regression of $F_{\text{IS}}$ on HIS1 comparison. Comparison of HIS1 results to their corresponding $F_{\text{IS}}$ results from simulated binned data that have had replicates with low HI1 variance removed. The $F_{\text{IS}}$ ranges were manipulated via ‘assortative mating’ and ‘selection’ scenario parameters shown in the methods section. The two panels show scenarios with differing population sizes in the simulations, indicated above in each panel. Blue line indicates a regression slope, the Red line indicates the expected 1:1 slope. In population sizes of 40, HIS1 showed an *r*‐squared of .749, *p*‐value = <.05 and RMSE = 0.304. In population sizes of 400, HIS1 showed an *r*‐squared of .769, *p*‐value = <.05 and RMSE = 0.340. In 400 population size scenarios, there was a reduced range of $F_{\text{IS}}$ values, from ~−0.5 to 1, whereas 40 population size scenarios showed the full range of $F_{\text{IS}}$ values from −1 to 1. Non‐binned data can be found in the supplement (Figure S10).

Results were analysed to investigate if we could come to the same conclusions about heterozygote deficits or excesses using a ^1^ *H*_IS_ value that we would using an *F*_IS_ value. Simulated results were analysed as a combined dataset (with all scenarios together, Figure 2), as well as when separated by different scenario parameters, such as the number of loci and allele distribution (Figures 3, 4, 5, 6). The comparison of ^1^ *H*_IS_ values with their corresponding *F*_IS_ values across the whole binned dataset showed a good regression fit (*R*‐squared of .756, *p* = <.001), close to the expected 45° line (Figure 2). Examining only simulations that altered the number of loci that were set in each simulation showed that ^1^ *H*_IS_ results performed well in all cases, but better with a larger number of loci (Figure 3; 3 loci: *R*‐squared of .445, *p*‐value = <.05; 5 loci: *R*‐squared of .452, *p*‐value = <.05; and 10 loci: *R*‐squared of .861, *p*‐value = <.05). Three‐locus scenarios only showed a range of *F*_IS_ values from ~ − 0.5 to 1, five‐locus scenarios from ~−0.5 to 1 and 10‐locus scenarios showed the full range from −1 to 1. Simulations given one of the two variant distribution scenarios showed that ^1^ *H*_IS_ performed well in both cases, but better in the ‘Uneven’ variant distribution scenario (Figure 4; Even: *R*‐squared of .593, *p*‐value = <.05; Uneven: *R*‐squared of .795, *p*‐value = <.05).

Simulation results were also analysed by separating data based on the demographic parameters: population size and generations of breeding. Simulations were set to run for one of the three generation times, giving other scenario parameters more time to affect the data. There was good regression fit in all cases, though slightly weaker with the longest generation time (Figure 5; 10 generations: *R*‐squared of .827, *p*‐value = <.05; 30 generations: *R*‐squared of .855, *p*‐value = <.05; and 50 generations: *R*‐squared of .723, *p*‐value = <.05). As generation time within each simulation increased, the number of replicates with low HI1 variance also increased. Simulations had two possible population sizes, which marginally influenced the accuracy of ^1^ *H*_IS_, and the range of values for ^1^ *H*_IS_ and *F*_IS_ (Figure 6). Note that in the larger population sizes, values tended to form clusters, which were related to initial values of variables other than population size: Small families simulated for 10 generations (*F*_IS_ ≈ 0.45 cluster); Small families simulated for 30 and 50 generations (*F*_IS_ ≥ 0.6 cluster); random mating and selection scenarios (*F*_IS_ = ~0 cluster).

### 3.2 Dolphin data

All values and results from the ^1^ *H*_IS_ calculations, along with *F*_IS_ results from the microsatellite data, are listed in Table 2. Shark Bay (SB) microsatellite data for the same population showed results that agree with the sign of our ^1^ *H*_IS_ method for *MHC I* in the same population. For SB, the positive ^1^ *H*_IS_ values suggest positive assortative mating or selection for homozygotes, which is consistent with the *F*_IS_, based on microsatellites. However, the ^1^ *H*_IS_ gave values an order of magnitude larger than *F*_IS_. Also at SB, the *F*_IS_ value of *MHC II* DQB showed a negative *F*_IS_ value, indicating a disagreement with the ^1^ *H*_IS_ results and *F*_IS_ from the microsatellites. For Bunbury (BB), the *MHC I* ^1^ *H*_IS_ results based on the average or mode locus‐number estimates are consistent with the microsatellite *F*_IS_ value in both direction and magnitude. However, the BB ^1^ *H*_IS_ value based on the One Individual locus‐number estimate is not comparable to the microsatellite *F*_IS_ value—both with respect to direction and magnitude (Table 2, Supplement S5).

**TABLE 2:** Heterozygote deficit or excess in *MHC I* and *MHC II* variants and microsatellites in dolphin populations —Locus‐number estimates, HI1¯ values, HS1 values, $E_{V}$ (evenness) values and ^1^ *H*_IS_ values for each population and locus‐number estimation method.

Population	MHC I sequences						Microsatellite data *F*_IS_ ^a^	MHC II DQB Single locus *F*_IS_
	Locus‐number estimation method (numbers refer to supplement S4)	Locus‐number estimate (non‐rounded)	HI1¯	^1^ *H*_S_	$E_{v}$	^1^ *H*_IS_		
Shark Bay (SB)	Mean (2)	6 (5.5)	1.787	2.371	0.746	0.540	0.0327	−0.024
	Median (3)	5 (4.5)				0.356		
	One Individual (1)	9 (8.5)				1.091		
Bunbury (BB)	Mean (2)	3 (3.3)	1.119	1.48	0.617	−0.015	−0.0376	NA
	Median (3)	3 (3.0)				−0.015		
	One Individual (1)	10 (10)				1.038		

^a^ *F*_IS_ values are estimated from microsatellite data from the same populations (Manlik, 2016; Manlik, Krützen, et al., 2019). Graphical representation of data is available in Supplement S5.

### 3.3 Penguin data

Major histocompatibility complex (MHC) sequence data were collected for three populations of little penguins (*Eudyptula minor*) in Western Australia (Vardeh, 2015). Results from ^1^ *H*_IS_ calculations, along with *F*_IS_ results from microsatellite data, are given in Table 3. Each individual penguin had relatively low diversity of variants (HI1¯ in Table 3). In contrast, the populations showed a relatively high amount of diversity of MHC variants across individuals (^1^ *H*_IS_ in Table 3). *F*_IS_ values based on microsatellite data agree with the sign of the MHC ^1^ *H*_IS_ values for the same population (Table 3, Supplement S5), and both estimates indicate a heterozygote deficit. Notably, results for the ALB and ESP populations gave ^1^ *H*_IS_ values that are at least an order of magnitude larger than *F*_IS_, although both *F*_IS_ and ^1^ *H*_IS_ suggested that the populations have a deficit of heterozygotes.

**TABLE 3:** Heterozygote deficit or excess of MHC and microsatellites in penguin populations —Locus‐number estimates, HI1¯ values, HS1 values, ^1^ *H*_IS_ and $E_{s}$ (evenness) values for each population and locus‐number estimation method.

Population	Locus‐number estimation method (numbers refer to supplement S4)	Locus‐number estimate (non‐rounded)	HI1¯	^1^ *H*_S_	$E_{v}$	^1^ *H*_IS_	*F*_IS_ (based on microsatellite data)^a^
Perth (PER)	Mean (2)	1 (1.36)	0.362	1.865	0.750	0.459	0.342
	Median (3)	1 (1)				0.459	
	One Individual (1)	4 (3.54)				2.27	
Albany (ALB)	Mean (2)	1 (1)	0.411	2.279	0.888	0.568	0.001
	Median (3)	1 (1.02)				0.568	
	One Individual (1)	3 (2.88)				2.02	
Esperance (ESP)	Mean (2)	1 (0.89)	0.246	2.345	0.889	0.702	0.093
	Median (3)	1 (1)				0.702	
	One Individual (1)	3 (3.22)				2.29	

*Note*: The MHC data were filtered to remove sequence reads that did not make up at least 10% of the sequence reads per individual.

^a^ *F*_IS_ values are estimated from microsatellite data from the same populations from Vardeh (2015). Graphical representation of data is available in Supplement S5.

## 4 DISCUSSION

*F*_IS_ is often used for the management and investigation of a population's assortative mating (including inbreeding) and selection on heterozygotes, so it will be useful that our ^1^ *H*_IS_ method can overcome the limitations of *F*_IS_ for multi‐gene families, such as MHC loci. On the basis of simulations of a wide range of scenarios, and analysis of real data of natural populations ^1^ *H*_IS_ showed a good relationship to *F*_IS_ (Figures 2, 3, 4, 5, 6; Tables 2 and 3), suggesting that ^1^ *H*_IS_ is a useful tool for analysing assortative mating and selection on heterozygotes, derived from data from multi‐locus gene families, in cases when conventional *F*_IS_ cannot be calculated.

We wish to distinguish our approach from those of some others, which differ from ours by omitting one or more of the following: (a) our focus on multi‐locus gene families that share multiple variant sequences at different locations in the genome, (b) our use of NGS data from non‐pooled individuals or (c) our estimation of statistics which can assess deficit/excess of heterozygotes relative to random mating and neutrality, akin to *F*_IS_ (see Table 1). For example, Schlötterer et al. (2014) explicitly called for the removal of copy‐number variants, which of course will exclude all multi‐locus gene families (such as MHC). Also, Ferretti et al. (2013) quite explicitly focused on pooled sequences of different individuals (which we do not), and assumed that all sequences can be mapped to a unique position in a reference genome; note that even if one had a reference genome, in a multi‐locus family that shares variant sequences between different positions in the genome, the correct location of variants would be difficult to ascertain, so that single‐locus statistics such as *F*_IS_ could not be calculated.

### 4.1 Simulations

Simulations showed that Equation 5 worked well under a wide variety of conditions. The fit between ^1^ *H*_IS_ and *F*_IS_ was good, irrespective of the number of loci and the evenness of variants (Figures 3 and 4). However, it is worth noting that in our simulations, the number of loci and the evenness of variants affected the range of values of ^1^ *H*_IS_ and *F*_IS_. When there were only three loci, both *F*_IS_ and ^1^ *H*_IS_ did not go below ~−0.5 (Figure 3). This may be due to the selection scheme in our simulation, which implemented selection only during the identification of individuals to be parents, and not through offspring survival; at the end of the simulation, the result was generated from a single generation of random mating without selection on the offspring, which would bring *F*_IS_ towards zero. Compared to scenarios with three loci, scenarios with ten loci would usually have a wider range of *F*_IS_ and ^1^ *H*_IS_ values, and so some ten‐locus replicates would maintain their *F*_IS_ and ^1^ *H*_IS_ values, whereas this is less likely to happen with three‐locus scenarios (Figure 3). The ^1^ *H*_IS_ to *F*_IS_ comparison showed a slightly more favourable regression result and lower RMSE when the variant distribution was ‘Uneven’, as well as showing a slightly better fit to the expected 1:1 (45°) regression line (Figure 4). This is likely partly due to ‘Uneven’ scenarios generating the full range of −1 to +1 *F*_IS_, whereas ‘Even’ scenarios rarely went below −0.5 *F*_IS_. This restricted range could be because uneven allele distribution may give a wider range of *F*_IS_ values, as a result of both $H_{e}$ and $H_{o}$ being very small, so that slight deviations could make a large change in Equation 5, resulting in the full range of values from −1 to +1. Figure 5 shows that the relationship between *F*_IS_ and ^1^ *H*_IS_ gave high *R*‐squared values for all generation times trialled, although slightly better at shorter generation times (30 and 10). However, it should be noted that despite the greater scatter, the departure from the expected 1:1 (45°) line decreased as generation time increased in Figure 5.

When the population size was 400, both *F*_IS_ and ^1^ *H*_IS_ values tended to cluster within scenarios (low variance of *F*_IS_ and ^1^ *H*_IS_ in ‘small families’ scenarios under different ‘generation time’ scenarios), as well as having a lower range of *F*_IS_ values (Figure 6). We believe this is due to the variance in population demographics being lessened in a larger population size (Hedrick, 1994). This would explain why scenarios with a population size of 400 scenarios clustered within scenarios and did not extend into more negative *F*_IS_ values.

An extreme result of drift is fixation of one or more loci. Population‐wide fixation is easy to detect without any sophisticated methods because there would be zero genetic diversity, so Hs1 and $H_{e}$ are zero, in which case ^1^ *H*_IS_ and *F*_IS_ are undefined (Table 4, first row). It is unlikely that a researcher would be interested in calculating either statistic from such data. A more subtle situation where ^1^ *H*_IS_ might give inaccurate results is when there is locus‐specific fixation, which occurs when each different locus is fixed for a different variant (Table 4, second row). When applied to a dataset with such a fixation pattern, *F*_IS_ would again be undefined, whereas ^1^ *H*_IS_ will give negative values. Because ^1^ *H*_IS_ is not locus‐specific, it will not allow detection of such a pattern of fixation and will instead lead to the interpretation that the individuals are maximally diverse, and the inference that some form of selection, assortative mating or demographic process is driving that diversity to give a negative ^1^ *H*_IS_ value. An extreme case of locus‐specific fixation (where every single locus is completely fixed across the population, as in Table 4, second row) can be detected by looking at variance of HI1 across the sample, because it will be zero in such a case. But the more subtle cases, in which, for instance half the loci are fixed, can be very difficult to detect, and would give ^1^ *H*_IS_ a negative bias on such datasets. While in our study we removed values with low variance of HI1, ^1^ *H*_IS_ did work well in some instances where HI1 was 0; or when *F*_IS_ = −1, and there was no variance of HI1 values between individuals (in these cases, ^1^ *H*_IS_ did tend to give values around the −0.9 range). However, these data, while showing correct results, were filtered out of our final dataset based on the criteria set out in the methods section (‘datum with no variation in the population’). This situation only occurred in ~0.003% of our simulations and seems to only represent the highly unusual case when every individual in the population (or sample) has exactly same heterozygote genotype.

**TABLE 4:** Two different scenarios for cases in which ^1^ *H*_IS_ would give inaccurate results.

Scenario	Genotype of every individual (with 4 loci)	^1^ *H*_IS_ result	$F_{\text{IS}}$ result
Total fixation	$\frac{C1}{C1};\frac{C1}{C1};\frac{C1}{C1};\frac{C1}{C1}$	Undefined	Undefined/0
Locus‐specific fixation	$\frac{C1}{C1};\frac{C2}{C2};\frac{C3}{C3};\frac{C4}{C4}$	−1	Undefined/0

There are several reasons for caution when estimating the number of loci, but there are appropriate steps that can be taken to help minimise these factors. First, results in Tables 2 and 3 show that the outcome depends upon the method used to estimate the number of loci for use in Equation 5. Second, NGS data also pose some problems with accuracy of our locus‐number estimation: due to the stochasticity of NGS, it is not always going to output the correct allele proportions needed to give an accurate estimate, especially for the ‘one individual’ method; therefore, it is usually more suitable to use the ‘mean’ or ‘median’ method to estimate the number of loci (Supplement S4). In the ‘Dolphin and Penguin Data’ section below, we always use the median, unless otherwise stated.

### 4.2 Dolphin and penguin data

Assessing the dolphin and penguin results is difficult, because there may be different selective pressures on MHC genes used to measure ^1^ *H*_IS_, compared to the microsatellites used to measure *F*_IS_. MHC genes have been reported to be under selection in many animals (Kloch et al., 2013; Sommer, 2005), whereas microsatellites are often selectively neutral. Therefore, the difference between ^1^ *H*_IS_ and *F*_IS_ results in Table 2 and Table 3 could be explained either by error or by differing selective pressures on the microsatellite and the MHC genes. Although selection for and against MHC heterozygotes at different loci is known to happen within a single species (Kloch et al., 2013), it is difficult to know what is the cause for the discrepancy between ^1^ *H*_IS_ and *F*_IS_ values because we cannot directly measure *F*_IS_ based on the MHC genes.

The BB dolphin population was relatively small at ~250 individuals (Manlik et al., 2016), but it is also known to have substantial immigration from other populations (Manlik et al., 2016). Thus, it is expected to have low inbreeding and *F*_IS_ of approximately zero, unless there are other effects such as selection. This accords with our finding that that both *F*_IS_ and ^1^ *H*_IS_ are close to zero (Table 2).

Shark Bay (SB) is a much larger population than BB, at ~3000 individuals (Manlik et al., 2016), however it is known that some inbreeding occurs in Shark Bay (Frère et al., 2010), so positive *F*_IS_ is expected, unless there is some other effect such as selection. Indeed, a positive $F_{\text{IS}}$ value was derived from the microsatellites. The MHC I ^1^ *H*_IS_ was also positive, but unlike at BB, ^1^ *H*_IS_ was much greater in magnitude compared to the microsatellite *F*_IS_ (Table 2). There are three possible explanations for this difference. First, the difference could be due to error of the ^1^ *H*_IS_ method, though the good agreement between MHC I ^1^ *H*_IS_ and microsatellite *F*_IS_ within BB suggests otherwise. Second, the difference between MHC I ^1^ *H*_IS_ and microsatellite *F*_IS_ may be due to misestimation of the number of loci. Manlik et al. (2016) was initially attempting to amplify a single MHC locus in MHC I by Sanger sequencing, but when using NGS confirmed that MHC I (exons 1 and 2) represented multiple loci. Possibly, we have overestimated the number of loci (note that the number of loci is lower in BB); this overestimation would have elevated our estimated value of ^1^ *H*_IS_. Third, there could possibly be selective effects acting on MHC I, which would have to be against MHC I heterozygotes to elevate the apparent heterozygote deficit in MHC I relative to microsatellites, which may only be responding to mild inbreeding. This interpretation requires further investigation to identify such selective pressures on MHC I in the SB population and the absence of such pressures in the BB population, possibly due to different histories—for example, a recent parasite infection can place selective pressures on MHC (Sommer, 2005). However, a selective interpretation for MHC I is strengthened by the disagreement of the two $F_{\text{IS}}$ values for SB: the microsatellite $F_{\text{IS}}$ and the MHC II $F_{\text{IS}}$ (Table 2). These values are likely due to different selective pressures acting on microsatellites and MHC II, with the microsatellites (and nearby linked genes) possibly being neutral, affected only by inbreeding, while the MHC II may have been subject to selection that favoured heterozygotes. Notably, MHC II DBQ nucleotide diversity and other diversity measures in SB are very high compared to BB (Manlik, Chabanne, et al., 2019), suggesting that there is some mechanism maintaining MHC II diversity in SB, either of which would be consistent with *MHC II* DBQ's negative $F_{\text{IS}}$ value in SB.

Compared to the dolphin data, the penguin data also showed that the excess of heterozygotes is consistent across the populations, and between the HIS1 and $F_{\text{IS}}$ values (Table 3), in all cases suggesting mild to strong inbreeding, or selection against heterozygotes, or possibly the Wahlund effect, due to the pooling of adjacent localities causing apparent depression of heterozygosity (Halliburton, 2004). The Average and Mode methods cannot have given overestimates of *L* because they both gave a value of unity, and indeed Vardeh (2015) was attempting to amplify a single locus of the gene family. However, other problems with this dataset may have impacted the results; for example, our analysis of the penguin dataset illustrates the limitation associated with the difficulty of identifying true singletons for the locus‐number estimation—as described above. We also tested methods from the literature that were designed to help with singleton estimation, however they were not useful in this case (Supplement S5).

## 5 CONCLUSIONS AND FURTHER OPTIONS

Though not addressed in our simulations, HIS1 might also be a useful tool for analysing heterozygote deficits or excesses in data from autopolyploid species, where $F_{\text{IS}}$ often cannot be applied for similar reasons. Additionally, in autopolyploids, other within‐population statistics that do not address heterozygote excess or deficit could possibly be calculated by an adaptation of the method of Ferretti et al. (2013). Some other applications of NGS to polyploids are not based on the within‐population questions we address, but on comparisons between species (McGrath, Gout, Doak, et al., 2014; McGrath, Gout, Johri, et al., 2014). In the future, it might also be possible to improve our work by using a modification of methods by Lynch et al. (2014) for estimation of allele frequencies from a genomic pool of individuals. However, the mathematics would have to be re‐worked extensively, to cope with three things: no pooling of individuals; multi‐locus gene families that share alleles, and focus on estimating $F_{\text{IS}};$ or a comparable statistic such as HIS1.

The use of HIS1 unlocks the potential for evolutionary and ecological studies investigating positive or negative assortative mating or selection, or other factors that affect heterozygote excess or deficit, using current and old datasets derived from multi‐locus gene families, especially of non‐model species. This can augment traditional $F_{\text{IS}}$ studies on single‐locus genes. Thus, multi‐locus gene family datasets can now be used to gain an understanding of positive or negative assortative mating or selective pressures on these extremely important gene families in wild populations. Such conclusions could not only give historical context to the populations studied, but also be used to guide future studies on related populations, especially in conservation applications. The power of HIS1 comes from four possibilities:

1. Researchers will be able to design studies that not only investigate the diversity in multi‐locus gene families, but potentially also infer assortative mating or selective pressures on those gene families, or other factors that affect heterozygote excess or deficit.
2. Researchers will be able to more directly study specific multi‐locus gene families that are known to have an impact on assortative mating and relative fitness of heterozygotes and homozygotes (such as MHC genes) and their population‐wide effects.
3. This method could also be applied retrospectively to datasets collected before that method existed, thus allowing researchers to utilise old MHC datasets to gain new insights into previously studied populations.
4. The new method is also directly applicable to cases where the entire genome is replicated, such as autopolyploidy.

## AUTHOR CONTRIBUTIONS

**Gabe D. O'Reilly:** Conceptualization (equal); formal analysis (equal); investigation (lead); methodology (lead); project administration (equal); writing – original draft (lead); writing – review and editing (equal). **Oliver Manlik:** Data curation (equal); formal analysis (equal); writing – review and editing (equal). **Sandra Vardeh:** Data curation (equal); formal analysis (equal); writing – review and editing (equal). **Jennifer Sinclair:** Data curation (equal); writing – review and editing (equal). **Belinda Cannell:** Data curation (equal); resources (equal). **Zachary P. Lawler:** Investigation (equal); writing – review and editing (equal). **William B. Sherwin:** Conceptualization (equal); methodology (equal); project administration (equal); supervision (lead); writing – original draft (equal); writing – review and editing (equal).

## CONFLICT OF INTEREST STATEMENT

There is no conflict of interest for this paper.

## Supporting information


Appendix S1.

## Data Availability

The simulation code to generate data, as well as data generated from our simulation run (which the results in this paper are based on) are available at the following GitHub repository: https://github.com/GabeDO/Detecting‐non‐random‐mating‐or‐selection‐in‐natural‐populations‐using‐multi‐locus‐gene‐families.
