# Supplementary material for: A new method for ecologists to estimate heterozygote excess and deficit for multi‐locus gene families
Source: Ecol Evol. 2024 Jul 23;14(7):e11561. doi: 10.1002/ece3.11561 (PMC11264353; doi:10.1002/ece3.11561)
Supplement: Supplementary file 1 — Appendix S1. [file ECE3-14-e11561-s001.docx]

# Supplement: Inference of assortative mating and selection on heterozygotes from multi-locus gene families.

## Supplement S1: Alternate estimation of selection or departure from random mating

Two other measures were tested called I’ (Equation S3) and O (Equation S4).

### MEASURE 1: $\boldsymbol{I}^{'}$

In the next generation sequencing output of each individual, for each locus, the homozygote loci each contribute one variant, while the heterozygote loci each contribute two variants. Some of these variants may be identical to variants contributed by other loci in the gene family. However, we start by assuming that all variants are distinguishable. We also started with the assumption that the population is in Hardy-Weinberg equilibrium (random mating, no selection, etc. so $F_{IS}=0)$. Then the expected number of variants per individual is:

$$I= \left( Homozygote contribution of 1 variant \right)+\left( Heterozygote contribution of 2 variants \right)= \sum_{l=1}^{L} \sum_{k=1}^{A_{l}} {P_{lk}}^{2}+2\sum_{l=1}^{L} (1-\sum_{k=1}^{A_{l}} {P_{lk}}^{2}) = 2L-\sum_{l=1}^{L} \sum_{k=1}^{A_{l}} {P_{lk}}^{2} [Equation S1]$$

$P_{lk}$ is the population proportion of allele $k$ (${1\leq k\leq A}_{l}$) at locus $l$ (${1\leq l\leq L}$) of the gene-family. Notice that this is now a function only of the expected number of homozygotes ${P_{lk}}^{2}$ and *L*, the number of loci (note that this method was trialed with *L* from main text – One individual, and with *L_2_* from supplement).

Next, we discarded the assumption that the population is in Hardy-Weinberg Equilibrium. When there is inbreeding or selection against heterozygotes ($F_{IS}\neq0$), the expected number of homozygotes at each locus, ${P_{lk}}^{2}$, is increased by addition of $P_{lk}\left( 1-P_{lk} \right)F_{is}$ (Falconer & McKay, 1996, Table 3.1)

So expected variants per individual:

$$I=2L-\sum_{l=1}^{L} \sum_{k=1}^{A_{l}} {\{P_{lk}}^{2}+P_{lk}\left( 1-P_{lk} \right)F_{is}\} [Equation S2]$$

Therefore with complete inbreeding, ($F_{IS}=1$), $I=L$, as expected, or with strong outbreeding, ($F_{is}=-1$), $I=2L$ if all $p$ values are 0.5. Thus, we can make a rescaled value that has approximately the same range as $F_{IS}$:

$I^{'}=\frac{3L-2I}{L} [Equation S3]$

This $I^{'}$ will show similar behaviour as for $F_{IS}$ , being approximately -1 when there is strong outbreeding, 0 when there is random mating, and +1 when there is complete inbreeding. Of course, the correspondence with $F_{IS}$ will be inexact if assumptions are violated, for example if some unknown proportion of the alleles at different loci are indistinguishable, and/or if some $p$ values are not 0.5.

### MEASURE 2: $\boldsymbol{O}$

This is simply $I$ scaled by the total number of variants in the population $V$

${O=\frac{I}{V} [Equation S4]}$

where expected $V= \sum_{l=1}^{L} A_{l}. O$ is expected to vary inversely with $F_{IS}$ , but it is difficult to give exact correspondence without knowing the $A_{l}$ values and other information.

These two measures were tested in the same way as ${}^{1}{H_{IS}}$ in the main text, with regression analysis and RMSE measurement on the binned datasets. *I’* was also tested using two locus-number estimation methods: Method 1 (One-individual) and Method 4 (Supplement S4, *L_2_*).

### Results:

*I’* showed poor R-squared values and poor RMSE results, when used with the locus-number estimation method described in the main text (One-individual; Fig S1). However, when applying *L_2_* (a method of estimating number of loci, defined in Supplement S4) *I’* performed better. While *I’* derived from Method 4 (Fig S2) did not surpass the${}^{1}{H_{IS}}$ results (Fig 2 in the main text), it did show a substantial improvement over *I’* using the main text locus-number estimation method (Method 1), giving an improved R-squared value and RMSE results. This is interesting because Method 4 had much more error when estimating the number of loci. *O* gave an R-squared value that was comparable to *I’*, but the RMSE value was much higher and the approach to 45° line was poor (Fig S3).


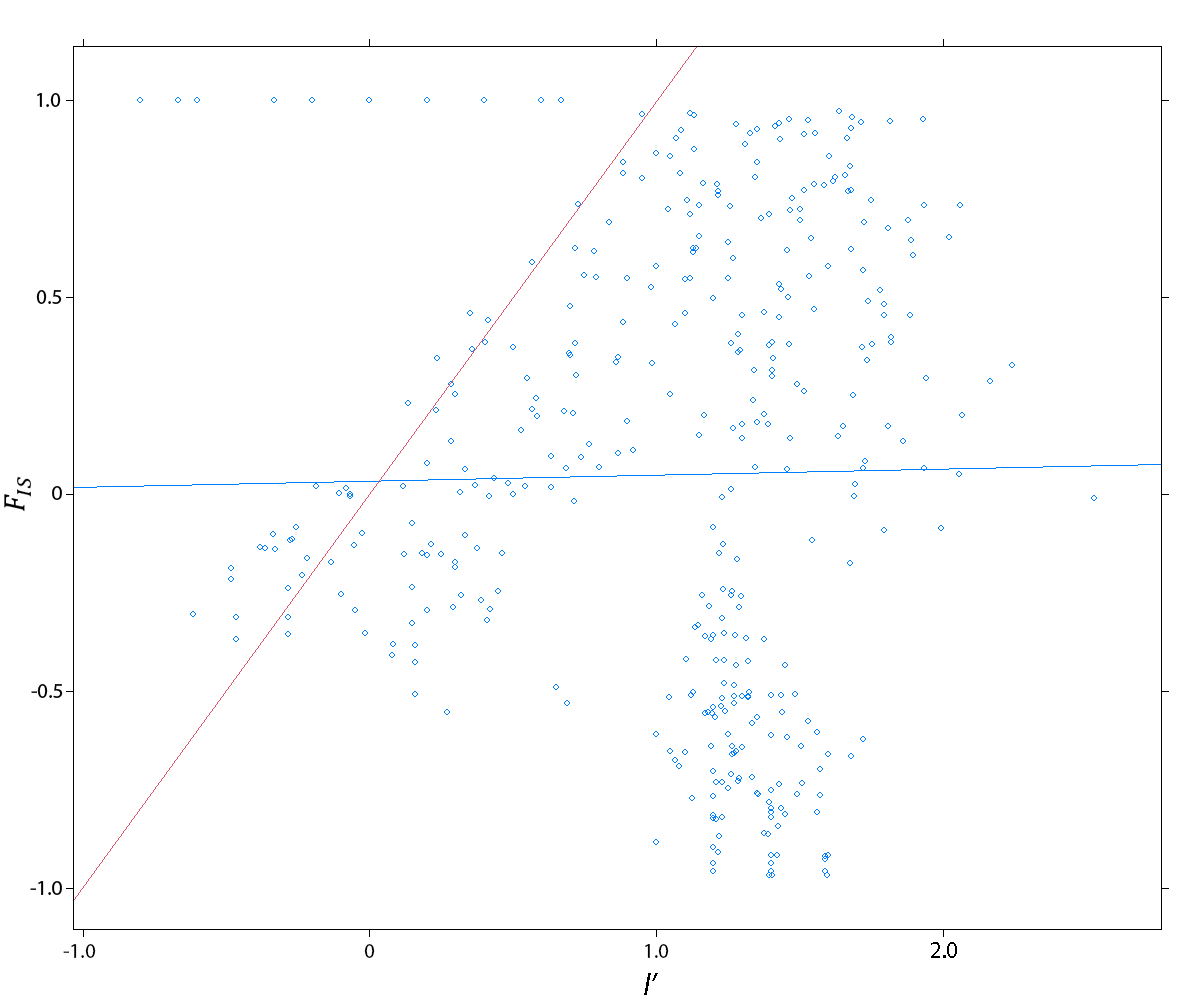


Fig S1: Regression of binned $F_{IS}$ on $I'$ (using ‘One-Allele’ locus-number estimation) from simulated data that has had replicates with no $I'$ variance removed. $F_{IS}$ ranges were manipulated via ‘mating’ and ‘selection’ treatment parameters shown in the methods section. Blue line indicates a regression slope, the Red line indicates the expected 1:1 slope. $I'$ showed an R-squared value of -0.00224, p-value = 0.742, RMSE = 1.3.


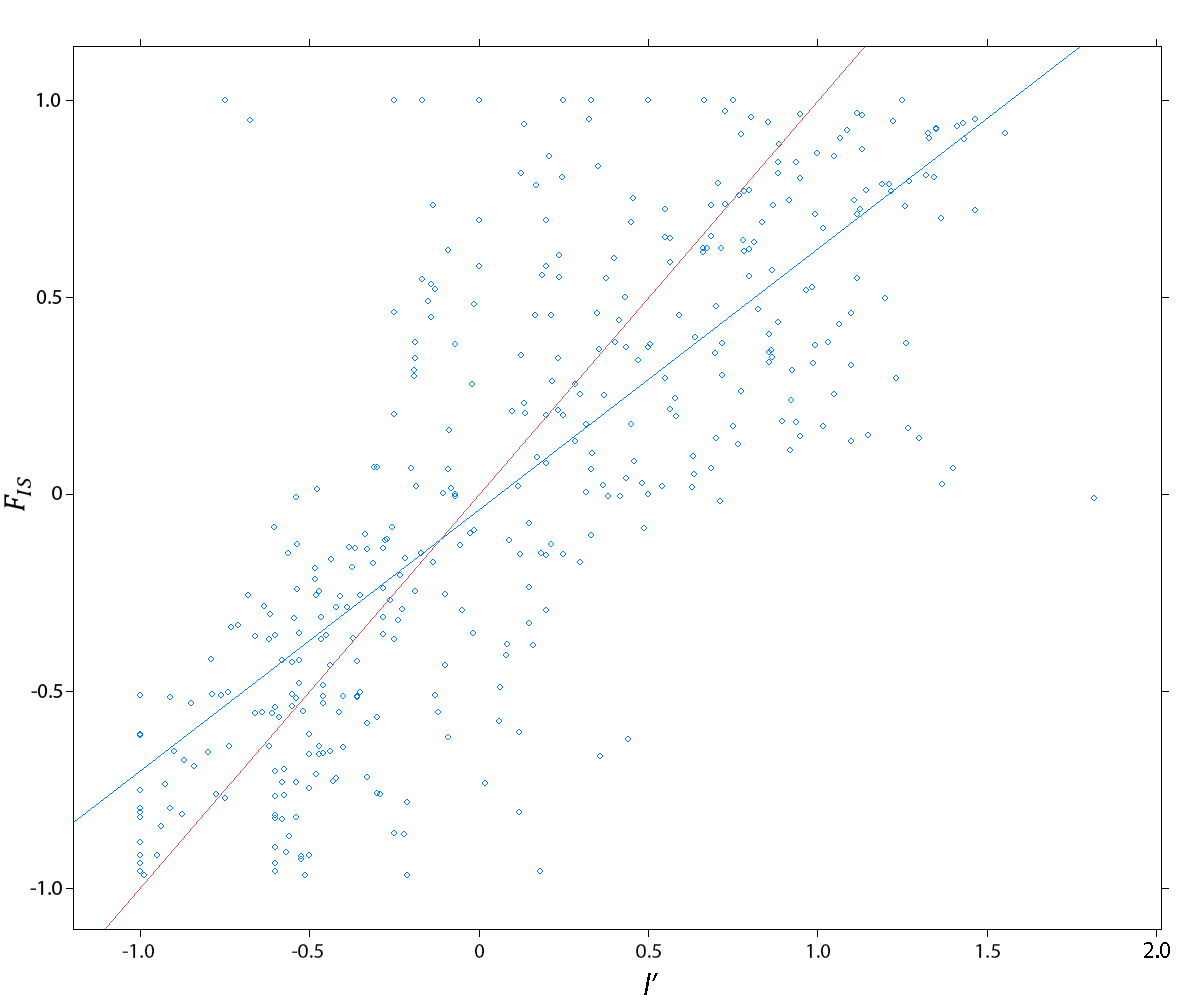
Fig S2: Regression of binned $F_{IS}$ on $I'$ (using *L_2_* locus-number estimation) from simulated data that has had replicates with no $I'$ variance removed. $F_{IS}$ ranges were manipulated via ‘mating’ and ‘selection’ treatment parameters shown in the methods section. Blue line indicates a regression slope, the Red line indicates the expected 1:1 slope. $I'$ showed an R-squared value of 0.580, p-value = < 0.05, RMSE = 0.444.


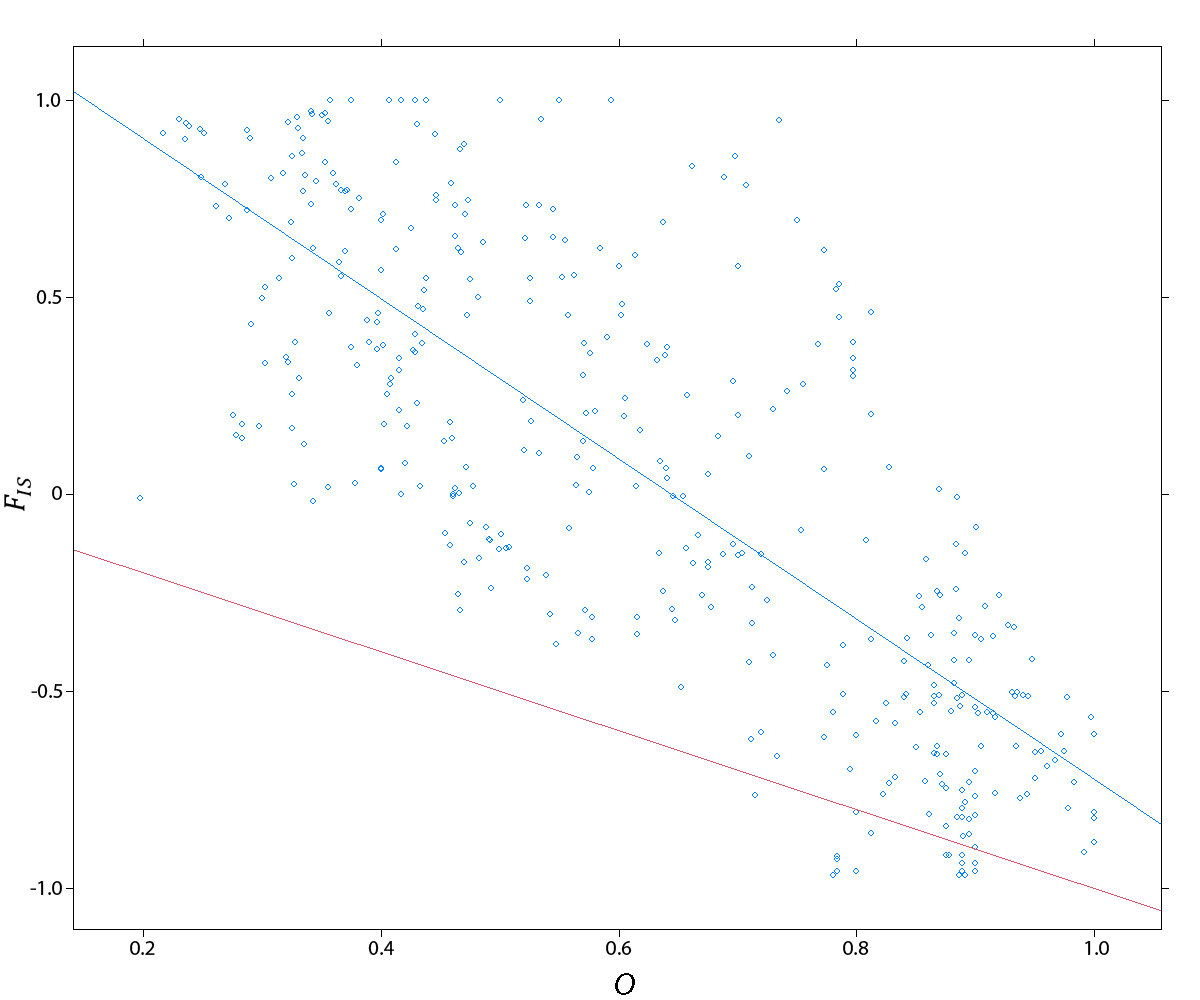


Fig S3: Regression of binned $F_{IS}$ against $O$ from simulated data that has had replicates with no $O$ variance removed. $F_{IS}$ ranges were manipulated via ‘mating’ and ‘selection’ treatment parameters shown in the methods section. Blue line indicates a regression slope, the Red line indicates the expected negative 1:1 slope. $O$ showed an R-squared value of 0.602, p-value < 0.05, RMSE = 0.951, with a negative slope, as expected.

## Supplement S2: Impact of read depth on our locus-number estimation method

Next generation sequencing (NGS) data is produced by a stochastic sampling process, during which variants detected in an individual are selected at random to be recorded. In single-locus sequences this is not an issue, because if the sequencing results show two variants, we know that there are two variants (i.e. alleles) at that locus. Moreover, if the sequencing only reveals one variant, the probability of bypassing an equally proportioned second variant is quite low, so we can be confident that the sample is homozygous for that locus (site). However, when dealing with large multi-locus gene families, there is an increased chance for the replicates in the sequencing sampling process (read depth) to either completely miss a variant or to sample some variants more than others despite equal proportions in the individual. This problem is exacerbated when the read depth is lower than the number of loci in the individual, because it will become impossible to sample every single variant (in their relative proportions, because there simply will not be enough replicates to represent each variant in an individual. In the context of our method, this would create problems with estimating number of loci. To further test the robustness of our “One Individual” locus-number estimation method (Supplement S4), some additional simulations were done with the final dataset stochastically sampling variants (based on their relative abundance) similarly to an NGS sampling process, with medium to low read depth, specified below. This code sampled alleles from each individual stochastically, because in NGS sequencing variants are read stochastically; the chance of a variant being read depends upon its frequency in the sample from that individual. We then applied our “One Individual” locus-number estimation (Supplement S4) to this new simulated NGS dataset to see how it would affect the accuracy of our locus-number estimation. This new dataset was reduced in scope compared to the main dataset used, by removing strong selection treatments. Our artificial read depth value was set to 30 for each individual. For real data, the higher the read depth the better, however 30 represents a high value that is also practical for most MHC researchers.

Assessment of${}^{1}{H_{IS}}$ on the simulated NGS dataset was analysed using the same methods as described in the main text for the full simulated dataset. The data filtering process was slightly different however, because replicates with low (< $1\times{10}^{-10}$) variance of richness were removed, as opposed to replicates with low (< $1\times{10}^{-10}$) variance of individual Shannon’s information (as was done for the complete simulated data). The rationale behind this was that due to the low read depth it was likely that some variants would not be sequenced.

These simulated NGS-like data increased the error of our locus-number estimates, and thus gave worse regression results than their non-NGS like counterpart in Fig 2 (Main text), though still with good R-squared and RMSE values (Fig S4).


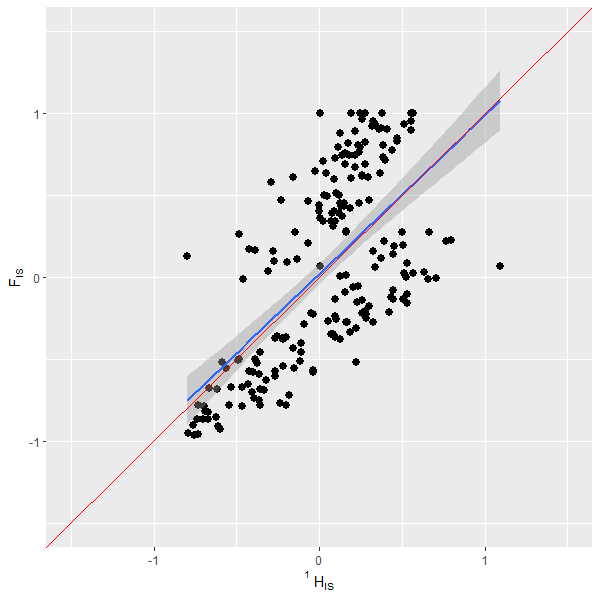
*Fig S4: Results of* $F_{IS}$ *regressed against* ${}^{1}{H_{IS}}$ *on binned NGS-like data. Comparison of* ${}^{1}{H_{IS}}$ *results to their corresponding binned* $F_{IS}$*results from simulated NGS data that has had replicates with low* ${}^{1}{H_{I}}$ *variance removed. The* $F_{IS}$*ranges were manipulated via ‘assortative mating’ and ‘selection’ treatment parameters shown in the methods section. Blue line indicates a regression slope, the Red line indicates the expected 1:1 slope.* ${}^{1}{H_{IS}}$ *showed an R-squared value of 0.407, p-value < 0.05, RMSE = 0.441.*

Additional simulations were also analysed on non-binned data. These simulated NGS-like data increased the error of our locus-number estimates, and thus gave worse regression results than its NGS-like binned data (Fig S5).


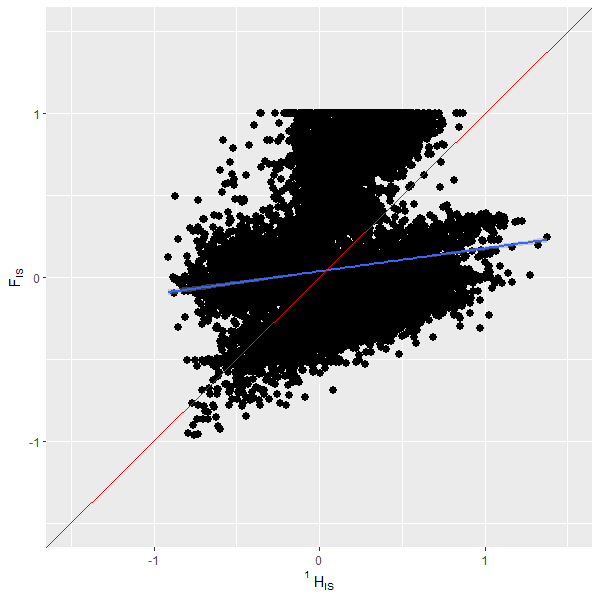


*Fig S5: Results of* $F_{IS}$ *regressed against* ${}^{1}{H_{IS}}$ *for NGS-like data. Comparison of* ${}^{1}{H_{IS}}$ *results to their corresponding non-binned* $F_{IS}$*results from simulated NGS data that has had replicates with low* ${}^{1}{H_{I}}$ *variance removed. The* $F_{IS}$ *ranges were manipulated via ‘mating’ and ‘selection’ treatment parameters shown in the methods section. Blue line indicates a regression slope, the Red line indicates the expected 1:1 slope.*${}^{1}{H_{IS}}$ *showed an R-squared value of 0.013, p-value = < 0.05, RMSE = 0.* *493.*

## Supplement S3: Regression results without data binning using Equation 5


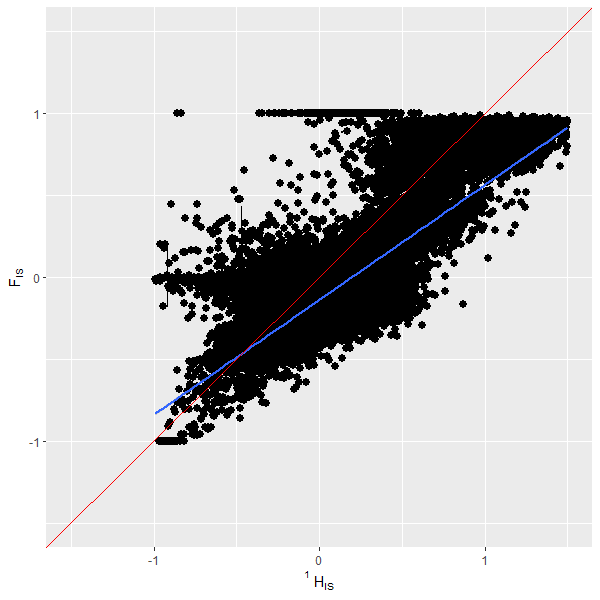


*Fig S6: Regression of* $F_{IS}$ *against* ${}^{1}{H_{IS}}$ *from simulated data that has had replicates with low* ${}^{1}{H_{I}}$ *variance removed.* $F_{IS}$*ranges were manipulated via ‘mating’ and ‘selection’ treatment parameters shown in the methods section. The total non-binned data, with all treatments together are shown. Blue line indicates a regression slope, the Red line indicates the expected 1:1 slope. Regression analysis gave an R-squared value of 0.756, p < 0.0* *01, and RMSE = 0.344. Corresponding binned data can be found in the main text (Fig 2).*


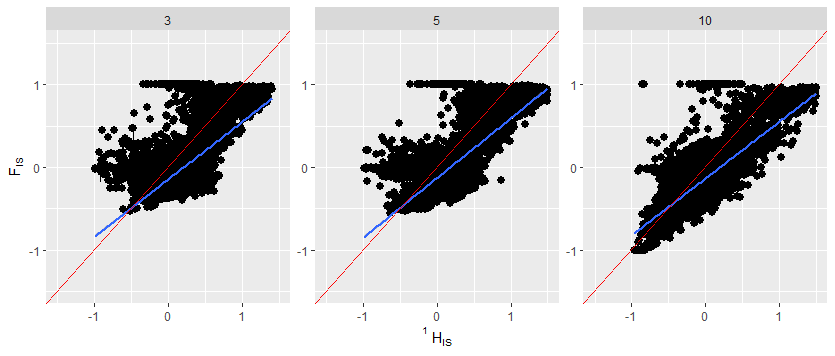
*Fig S7: How number of loci affects regression of*$F_{IS}$ *on* ${}^{1}{H_{IS}}$*. Comparison of* ${}^{1}{H_{IS}}$ *results to their corresponding* $F_{IS}$ *results from simulated non-binned data that has had replicates with low* ${}^{1}{H_{I}}$ *variance removed. The* $F_{IS}$*ranges were manipulated via ‘mating’ and ‘selection’ treatment parameters shown in the methods section**. The three panels show treatments with differing numbers of loci set up in the simulation, indicated above each panel. Blue line indicates a regression slope, the Red line indicates the expected 1:1 slope. In treatments with 3 loci,* ${}^{1}{H_{IS}}$ *showed an R-squared of 0.452, p-value = < 0.05, and RMSE = 0.364. In treatments with 5 loci,* ${}^{1}{H_{IS}}$ *showed an R-squared of 0.600, p-value = < 0.05, and RMSE = 0.351. In treatments with 10 loci,* ${}^{1}{H_{IS}}$ *showed an R-squared value of 0.726 , p-value < 0.05, and RMSE = 302. 3 Loci treatments only showed a range of* $F_{IS}$ *values from ~-0.5 to 1, 5 loci treatments from ~-0.5 to 1, and 10 loci treatments showing the full range of -1 to 1. Corresponding binned data can be found in the main text (Fig 3).*


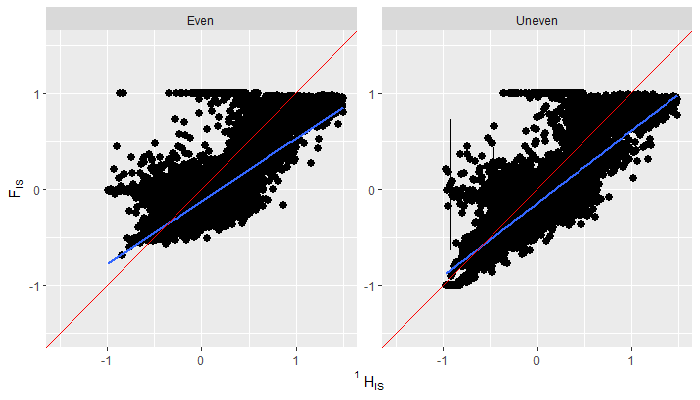

*Fig S8: How allele distribution affects regression of*$F_{IS}$ *on* ${}^{1}{H_{IS}}$*. Comparison of* ${}^{1}{H_{IS}}$ *results to their corresponding* $F_{IS}$ *results from non-binned simulated data that has had replicates with low* ${}^{1}{H_{I}}$ *variance removed. The* $F_{IS}$*ranges were manipulated via ‘mating’ and ‘selection’ treatment parameters shown in the methods section. The two panels show treatments with differing distribution of variants in the simulation, indicated above each panel. Blue line indicates a regression slope, the Red line indicates the expected 1:1 slope. In treatments with an Even variant distribution,* ${}^{1}{H_{IS}}$ *showed an R-squared of 0.601, p-value = < 0.05, and RMSE = 351. In treatments with an Uneven variant distribution,* ${}^{1}{H_{IS}}$ *showed R-squared of 0.496, p-value = < 0.05, and RMSE = 0.337.* ${}^{1}{H_{IS}}$ *in Even treatments, showed a reduced range of* $F_{IS}$ *values, from -0.5 to 1, whereas Uneven treatments showed the full range of* $F_{IS}$ *values from -1 to 1. Corresponding binned data can be found in the main text (Fig 4).*


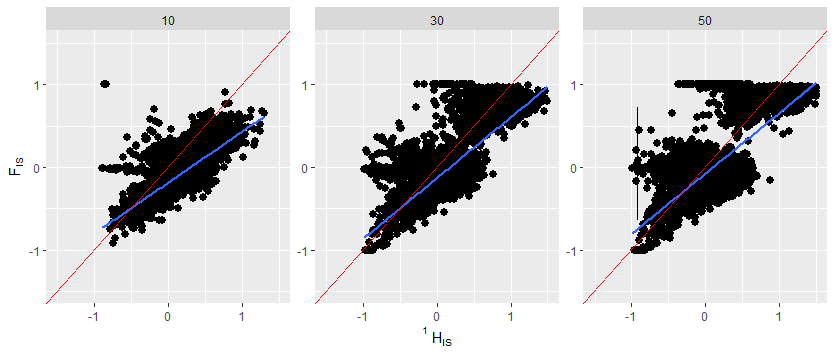
*Fig S9: How number generations simulated affects regression of*$F_{IS}$ *on* ${}^{1}{H_{IS}}$*. Comparison of* ${}^{1}{H_{IS}}$ *results to their corresponding* $F_{IS}$ *results from non-binned simulated data that has had replicates with low* ${}^{1}{H_{I}}$ *variance removed. The* $F_{IS}$*ranges were manipulated via ‘mating’ and ‘selection’ treatment parameters shown in the methods section. The three panels show treatments with differing numbers of generations simulated, indicated above each panel. Blue line indicates a regression slope, the Red line indicates the expected 1:1 slope. Ten generation data having an r-squared of 0.627, p-value = < 0.05 and RMSE = 0.342. Thirty generation data had an r-squared of 0.663, p-value = < 0.05, and RMSE = 0.324. Fifty generation data had an r-squared of 0.577, p-value = < 0.05, and RMSE = 0.362. Corresponding binned data can be found in the main text (Fig 5).*


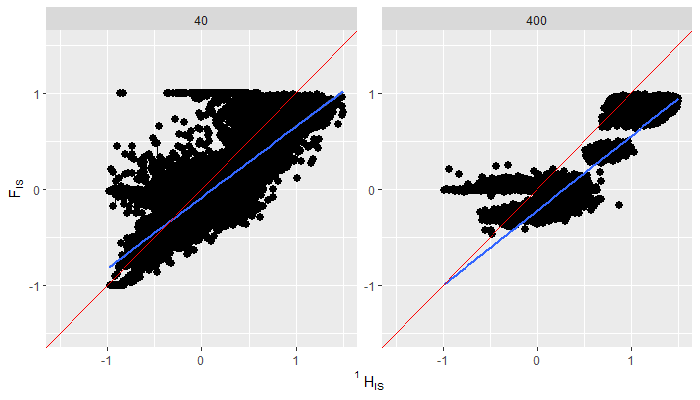

*Fig S10: How population size affects regression of*$F_{IS}$ *on* ${}^{1}{H_{IS}}$*. Comparison of* ${}^{1}{H_{IS}}$ *results to their corresponding* $F_{IS}$ *results from non-binned simulated data that has had replicates with low* ${}^{1}{H_{I}}$ *variance removed. The* $F_{IS}$ *ranges were manipulated via ‘mating’ and ‘selection’ treatment parameters shown in the methods section. The two panels show treatments with differing population sizes in the simulations, indicated above each panel. Blue line indicates a regression slope, the Red line indicates the expected 1:1 slope. In population sizes of 40,* ${}^{1}{H_{IS}}$ *showed an r-squared of 0.495, p-value = < 0.05, and RMSE = 0.324. In population sizes of 400,* ${}^{1}{H_{IS}}$ *an r-squared of 0.771, p-value = < 0.05, and RMSE = 0.363.* ${}^{1}{H_{IS}}$ *in 400 population size treatments, showed a reduced range of* $F_{IS}$ *values, from ~-0.5 to 1, whereas 40 population size showed the full range of* $F_{IS}$ *values from -1 to 1. Corresponding binned data can be found in the main text (Fig 6).*

## Supplement S4: Estimation of number of loci

It is common that the exact number of loci (“*L*”) is not known when studying a multi-locus gene family in a non-model organism. If there are no prior data on a study population, it may be possible to estimate the number of loci by examining other research in similar organisms or multi-locus gene families; however, these approaches may not be helpful when dealing with a novel species, or because the number of MHC loci can even vary within species (Bowen *et al,* 2004; Siddle *et al*, 2010).

For cases where no reasonable estimate for the number of loci can be made from other data, this section (S4) presents four ways to estimate the minimum number of loci, if certain assumptions are made. Note, however, that we would not recommend our methods (especially the ‘one-individual’ method) as a substitute to a robust independent investigation of number of loci in a species.

First method: In the first 'one individual' method to estimate the number of loci, we assume that at least one individual, which has been sampled from the population, has a true singleton (ie, an allele represented only once, at only one of the loci). It then becomes possible to calculate the minimum number of loci. The closer the sample data comes to fulfilling this assumption, the more accurate the estimation of number of loci will be. The data set will give relative proportions of variants over all loci as ratio, with the assumed singleton being “1” in that ratio. For example, the output for an individual with three variants (variants *B1*, *B2* and *B3*) might be presented as the ratio: 1:3:2, indicating that there are three times as many *B2* variants than *B1* variants, and two times as many *B3* variants than *B1* variants. If we assume that this individual has only one *B1* variant (ie the individual is heterozygous for the *B1* variant at a single locus), the sum of this ratio (1+3+2) would be twice the minimum number of loci (because each locus contains two alleles). In this case 2*L* ≥ 6, so there are at least *L*≥3 loci. The highest value for 2 *L* of all the individuals, is divided by two and rounded up to the next integer to gain a minimum estimate of number of loci. With very high read depth, whichever individual gives the biggest sum of the ratio will provide the most accurate estimate of the minimum number of loci, which will be the assumption for this 'one individual' method (Table S1).

|  | Variants: | | | | Sum | Estimated number of loci |
| --- | --- | --- | --- | --- | --- | --- |
|  | *B1* | *B2* | *B3* | *B4* |  |  |
| Individual 1: | 1 | 1 | 2 | 1 | =5 | 3 |
| Individual 2: | 1 | 4 | 3 | 2 | =10 | 5 |
| Individual 3: | 1 | 0 | 1 | 0 | =2 | 1 |
| Individual 4: | 1 | 0 | 1 | 3 | =5 | 3 |

*Table S1: Example data set on a population with four individuals showing relative proportions of variants as ratios. The ratios are adjusted so that the least frequent variant is represented by “1”, then summed for each individual, the highest value among these summed values is rounded to the next even number then divided by two to gain a minimum estimate of number of loci. In the data shown for individual 1, five is divided by two then rounded up to give minimum L ≥ 3. This rationale is explained in the text of S4.*

It is common for NGS datasets to be formatted in number of sequence reads rather than ratios. In this case, for each individual simply divide each number of sequence reads per variant by the lowest value of number of sequences reads for any variant (post filtering), then round up any decimals to a whole number. This should result in ratios which can then be used for the above method to get a minimal estimate of number of loci. For example, if the read numbers for variants B1 B2 and B3 were 12, 35, 23 respectively, then divide the values by 12 (the lowest read value) to get 1:2.9:1.9, sum those to get 5.8 and round to the nearest integer, giving 2*L* = 6, meaning there is a minimum of three loci. This can be expressed by the following equation:

$$L=round\left( \frac{\sum_{i=1}^{V} \frac{r_{i}}{r_{min}}}{2} \right) [Equation S6]$$

Where *r* is the number of sequence reads, $r_{min}$ is the minimum number of sequence reads for an individual, and the other symbols the same as in Equation 5; ‘round' indicates that the value is rounded to the nearest integer.

Similar methods have been applied previously, although they use presence/absence of alleles, rather than attempting to quantify abundance using sequence reads as our method does, and gave less accurate estimates of the number of loci in our simulations, where the number of loci L was known (Babik *et al,* 2009; Heimeier *et al* , 2009 ; Sommer *et* al, 2013). Some methods have also identified some loci as fixed in order to get better estimations alongside presence /absence methods (Stervander *et al*, 2020), and other methods have taken a computational approach to genotyping MHC (Stuglik *et al*, 2011).

Of course, with relatively low read-depth in real data, sequence misreads, or missing data might lead to inaccuracy of the 'One Individual' method for estimating *L* the number of loci. Therefore, other methods are needed.

Method two: The ‘Mean’ method for estimating the number of loci, which takes the mean estimate of *L* over all individuals, to minimise the impact of any sequence read errors greatly inflating the value of *L*. The ‘Mean’ locus-number estimation methods will help when the data has sequence misreads or missing data, but it may not completely overcome more systematic issues to do with NGS data, such as low read depth or NGS’s inherent stochasticity in which variants it sequences (Smith & Peay, 2014; Qin *et al*, 2016), or other inherently random processes throughout genetic sequencing and data collection. The impact of NGS data’s stochasticity was stated in Supplement S2.

Method three: The ‘Median’ method for estimating the number of loci, which takes the median estimate of *L* over all individuals, again to minimise the impact of any sequence read errors greatly inflating the value of *L*. The “Median’ methods has similar limitations to the ‘Mean’ method.

Method four: One other locus-number estimation method was developed and tested, called $L_{2}$ (Equation S7). $L_{2}$ was equal to the maximum number of variants held in any individual, then divided by two and rounded up. The logic for $L_{2}$ is that: if we assume that there is an individual in the population that has two unique variants at each locus, then the maximum number of variants held in that individual will be equal to double the number of loci. Because each locus contains two alleles, if this maximum value is an odd number, when divided by two it will result in a decimal. This is then rounded up because loci do not naturally occur in fractions. $L_{2}$ did not perform as well as the method presented in the main text, when tested on simulated data where the true number of loci was known. $L_{2}$ tended to underestimate the number of loci present in the population (Fig S11).

$$L_{2}= \left[ \frac{Max number of variants in an individual}{2} \right] [Equation S7]$$

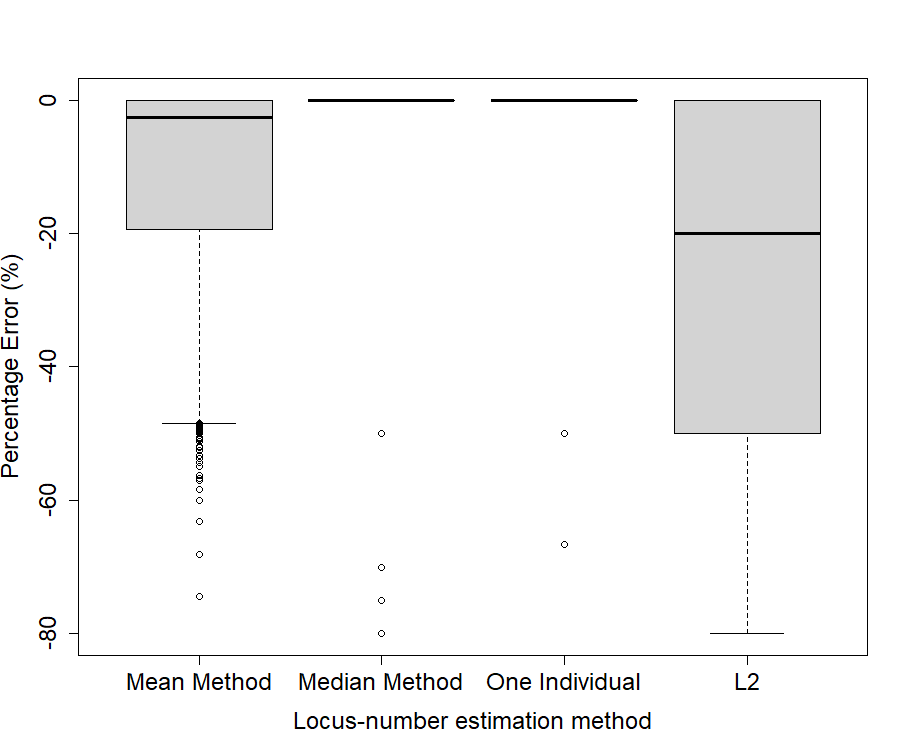


Fig S11: Percentage error of different locus-number estimation methods: One Individual, Mean, Median, and Method 4, $L_{2}$ from Equation S7. Showing the median (the thick bar), upper and lower quartile (the grey box), the lowest non-outlier observations (the whisker) and outliers (the dots). This Fig uses the same binned simulation data described in the main text.

Tables 2 and 3 in the main text show that the "one individual" method for estimation of number of loci, which assumes that the sample will have at least one individual possessing a singleton variant, gives extremely high values, resulting in unrealistically high values of ${}^{1}{H_{IS}}$. Therefore, use of this method can cause Equation 5 to be inaccurate in situations where the data would be unlikely to have singletons, such as a population or sample with few, but equally abundant variant sequences and many loci. This makes sense, as it is less likely for any individual to have a singleton variant, if their genotype is dominated by 1 or 2 variants across many loci. Thus, it is usually more suitable to use the "mean" or "median" method to estimate the number of loci. For microsatellites and MHC I the direction of departure is for a slight excess of heterozygotes - positive ${}^{1}{H_{IS}}$ and $F_{IS}$ values. The only exception to this is when using the One-Individual estimate for number of loci in the BB dolphin population, which gave a positive ${}^{1}{H_{IS}}$ value. This accords with our suggestion that the One-Individual method of locus-number estimation is not suitable unless the sequencing depth is very high. The One-Individual locus-number estimation appears to be unsuitable for this relatively low-depth sequencing, giving ${}^{1}{H_{IS}}$ values well above the expected maximum of +1. Such elevation of ${}^{1}{H_{IS}}$ is expected if *L* is over-estimated.

## Supplement S5: Graphical representation of penguin and dolphin data


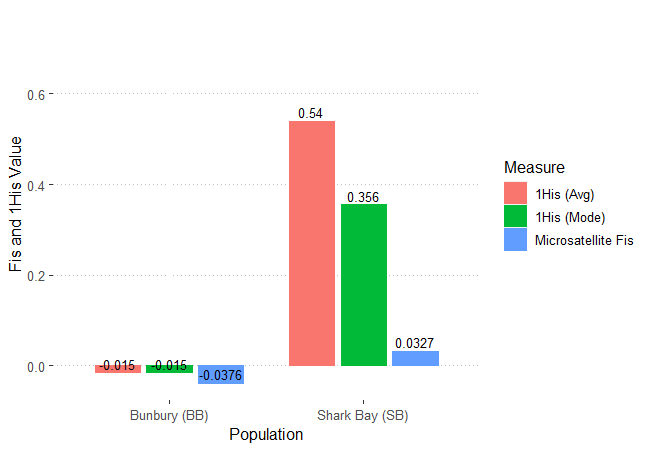


Fig S12: A histogram showing the following measures for the two dolphin populations using different methods for estimating number of loci (Supplement S4): ${}^{1}{H_{IS}}$ using the ‘average’ locus-number estimate; ${}^{1}{H_{IS}}$ using the ‘mode’ locus-number estimate; $F_{IS}$ using microsatellite data from multiple loci. ${}^{1}{H_{IS}}$ using the ‘one individual’ loci estimate is not included in the histogram, because the values exceeded 1.


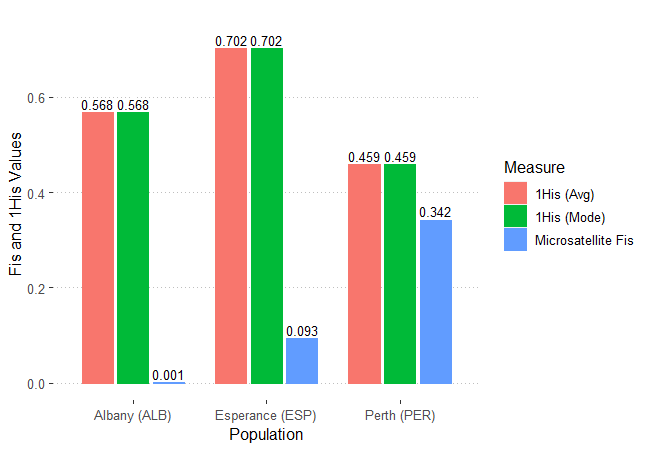


Fig S13: A histogram showing the following measures for the three penguin populations using different methods for estimating number of loci (Supplement S4): ${}^{1}{H_{IS}}$ using the ‘average’ locus-number estimate; ${}^{1}{H_{IS}}$ using the ‘mode’ locus-number estimate; $F_{IS}$ using microsatellite data from multiple loci. ${}^{1}{H_{IS}}$ using the ‘one individual’ locus-number estimate is not included in the histogram, because the values exceeded 2.

## Supplement S6: Missing singleton data correction

The possibility of missing data was investigated, because the values used in calculating ${}^{1}{H_{IS}}$ would be affected if the sequencing missed singletons. If there were singleton variants in the population that sequencing did not pick up: $E_{V}$ would be overestimated; L would potentially be underestimated; and ${}^{1}{H_{S}}$ and ${}^{1}{H_{I}}$would be underestimated. This would lead to an inaccurate ${}^{1}{H_{IS}}$ calculation. To counteract this effect of missing singleton values, we tested a mathematical correction from Chao & Jost 2015. This method was applied to some individuals in the penguin data to see if it gave realistic results on a real dataset. Unfortunately, the results it gave did not seem to be realistic. The correction estimated that on average, each individual had 316 missing singleton variants, with a maximum of 2654 missing singleton variants. These estimates do not seem realistic, especially because they would substantially inflate the locus-number estimates using the methods outlined in the main text. These large numbers could be due to the large number of variant sequences in the penguin data, especially before filtering is taken into account, possibly due to sequencing errors that unfortunately abound in NGS data.

## Supplement S7: Chao equations

There have been two equations developed that make an approximate connection between Shannon entropy (part of the calculation for ${}^{1}{H_{is}}$ in equation 4a or 4b) with Hardy-Weinberg expected heterozygosity (part of the calculation for $F_{IS}$) under two mutation models for a single locus (Chao et al, 2015). However, these equations cannot be used in the current context, because they only make a connection between one component of $F_{IS}$, the $H_{e}$ at single loci, and one component of ${}^{1}{H_{is}}$, the ${}^{1}H$ contributions of each locus at the population level. These ${}^{1}H$ contributions underly the ${}^{1}{H_{s}}$ value in a way that can only be determined if the exact pattern of variant sharing between loci is known. However, the added value of our work is that it deals with cases where this pattern of variant sharing is not known, as is usually the case in NGS data for multi-locus gene families. Therefore, the Chao et al (2015) equations cannot be used because extra assumptions required to fill in the remaining components of ${}^{1}H$ is in equation 4a or 4b make the applicability very limited.

There are no trials associated with this analysis, because Chao et al’s the equations cannot be used without information about which variants are alleles at which loci.

## References

Babik, W., Taberlet, P., Ejsmond, M. J., & Radwan, J. (2009). New generation sequencers as a tool for genotyping of highly polymorphic multi-locus MHC system. *Molecular Ecology Resources*, *9*(3), 713-719.

Bowen, L., Aldridge, B. M., Gulland, F., Van Bonn, W., DeLong, R., Melin, S., ... & Johnson, M. L. (2004). Class II multiformity generated by variable MHC-DRB region configurations in the California sea lion (Zalophus californianus). Immunogenetics, 56(1), 12-27.

Chao, A., Jost, L., Hsieh, T. C., Ma, K. H., Sherwin, W. B., & Rollins, L. A. (2015). Expected Shannon entropy and Shannon differentiation between subpopulations for neutral genes under the finite island model. PloS one, 10(6), e0125471.

Falconer, D. S. (1996). Introduction to quantitative genetics. Pearson Education India.

Heimeier, D., Baker, C. S., Russell, K., Duignan, P. J., Hutt, A., & Stone, G. S. (2009). Confirmed expression of MHC class I and class II genes in the New Zealand endemic Hector's dolphin (Cephalorhynchus hectori). Marine Mammal Science, 25(1), 68-90.

Qin, L. X., Tuschl, T., & Singer, S. (2016). Empirical insights into the stochasticity of small RNA sequencing. Scientific Reports, 6(1), 1-8.

Siddle, H. V., Marzec, J., Cheng, Y., Jones, M., & Belov, K. (2010). MHC gene copy number variation in Tasmanian devils: implications for the spread of a contagious cancer. Proceedings of the Royal Society B: Biological Sciences, 277(1690), 2001-2006.

Smith, D. P., & Peay, K. G. (2014). Sequence depth, not PCR replication, improves ecological inference from next generation DNA sequencing. PloS one, 9(2), e90234.

Sommer, S., Courtiol, A., & Mazzoni, C. J. (2013). MHC genotyping of non-model organisms using next-generation sequencing: a new methodology to deal with artefacts and allelic dropout. BMC Genomics, 14(1), 1-17.

Stervander, M., Dierickx, E. G., Thorley, J., Brooke, M. D. L., & Westerdahl, H. (2020). High MHC gene copy number maintains diversity despite homozygosity in a critically endangered single‐island endemic bird, but no evidence of MHC‐based mate choice. Molecular Ecology, 29(19), 3578-3592.

Stuglik, M. T., Radwan, J., & Babik, W. (2011). jMHC: Software assistant for multi-locus genotyping of gene families using next‐generation amplicon sequencing. Molecular ecology resources, 11(4), 739-742.
